# A causal role for frontal cortico-cortical coordination in social action monitoring

**DOI:** 10.1038/s41467-020-19026-y

**Published:** 2020-10-16

**Authors:** Taihei Ninomiya, Atsushi Noritake, Kenta Kobayashi, Masaki Isoda

**Affiliations:** 1 Division of Behavioral Development, Department of System Neuroscience, National Institute for Physiological Sciences, National Institutes of Natural Sciences, 38 Myodaiji, Okazaki, Aichi 444-8585 Japan; 2 Department of Physiological Sciences, School of Life Science, The Graduate University for Advanced Studies (SOKENDAI), Hayama, Kanagawa 240-0193 Japan; 3 Section of Viral Vector Development, National Institute for Physiological Sciences, National Institutes of Natural Sciences, 38 Myodaiji, Okazaki, Aichi 444-8585 Japan

**Keywords:** Social neuroscience, Neurophysiology

## Abstract

Decision-making via monitoring others’ actions is a cornerstone of interpersonal exchanges. Although the ventral premotor cortex (PMv) and the medial prefrontal cortex (MPFC) are cortical nodes in social brain networks, the two areas are rarely concurrently active in neuroimaging, inviting the hypothesis that they are functionally independent. Here we show in macaques that the ability of the MPFC to monitor others’ actions depends on input from the PMv. We found that delta-band coherence between the two areas emerged during action execution and action observation. Information flow especially in the delta band increased from the PMv to the MPFC as the biological nature of observed actions increased. Furthermore, selective blockade of the PMv-to-MPFC pathway using a double viral vector infection technique impaired the processing of observed, but not executed, actions. These findings demonstrate that coordinated activity in the PMv-to-MPFC pathway has a causal role in social action monitoring.

## Abstract (web summary)

Social interactions require monitoring others’ actions to optimally organise one’s own actions. Here, the authors show that the pathway from the ventral premotor cortex (PMv) to the medial prefrontal cortex (MPFC) is causally involved in monitoring observed, but not executed, actions.

## Introduction

Primates, including humans, are social creatures and coordinate their behavior to maintain group cohesion. Successful social exchanges require monitoring others’ actions to optimally organize one’s own actions. This social action monitoring for response optimization has been thought to be mediated at the frontal level by brain regions centered in the ventral premotor cortex (PMv) and the medial prefrontal cortex (MPFC)^1–3^. The PMv is a cortical area in which the so-called mirror neurons were discovered in the macaque^4^. This class of neurons discharge not only when executing specific actions, but also during the observation of similar actions performed by others. This shared coding of actions between the self and others has been associated with diverse roles, including intention reading via embodied simulation^5^, and is hypothesized to be deficient in autism spectrum disorder (ASD)^6–8^. Later work has shown, however, that the MPFC also contains neurons with mirror properties^9–11^. In a similar vein, it has been argued that the existence of self neurons and other neurons in the MPFC^9–11^ may characterize its unique role in self-other distinction^12^. Self neurons and other neurons are those that selectively or preferentially encode the self-action and others’ action, respectively, and the paucity of other neurons in the MPFC is associated with a spontaneous autistic phenotype in a macaque monkey^13^. However, such separate neuronal coding of self and other is not confined to the MPFC. Although studies on the PMv have usually emphasized the existence of motor neurons and mirror neurons, a subset of PMv neurons, albeit in the minority (~5%), are purely action observation neurons^14^. These findings suggest that the existence of a particular class of neurons per se cannot uniquely define a particular area in the brain. Despite a growing interest in cortical mechanisms of social action monitoring, the similarities and differences between the PMv and MPFC have yet to be determined. This is in part because direct comparisons of neuronal activities have not been performed between the two areas in the same monkeys performing the same task.

In relation to the issue raised above, there has been much debate on the functional organization of the PMv and MPFC. First, it is controversial whether the two cortical areas show a preference for actions of biological agents. Some studies show that the PMv is activated by actions performed by biological, but not nonbiological, entities^4,14–16^, while others show the lack of such biological preference^17,18^. There is even evidence that mirror neurons in the PMv can become active in the absence of any visible actions^19,20^. In the MPFC, activation is selective for biological interactions as assessed using functional neuroimaging methods^3^; however, this issue has never been addressed systematically at the single-neuron level.

Second, it is highly controversial whether the PMv and MPFC coordinate with one another for social information processing. A large body of work in human neuroimaging has shown that the two areas are rarely concurrently active^1^, leading to the hypothesis that their roles in social cognition are mutually independent or even divergent^21,22^. This is obviously a puzzle, however, if one considers ample evidence showing rich anatomical connections between the PMv and MPFC^23–28^. In particular, stronger tuning to the self and others’ actions in more caudal MPFC divisions is associated with stronger connections with the PMv^28^. Although several neuroimaging and theoretical studies^29–36^ suggest possible interactions between the PMv and MPFC, or between neural systems to which these areas are thought to belong separately, this issue has never been addressed electrophysiologically in the macaque.

**Fig. 1:**
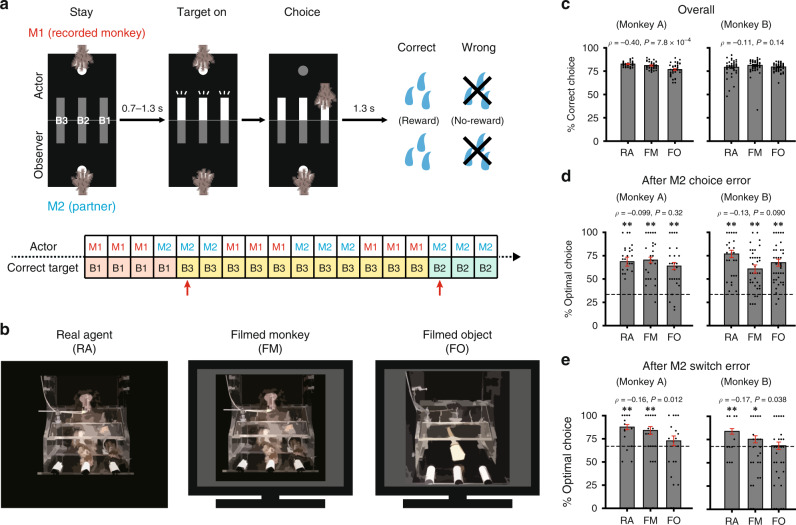
Behavioral task and performance. **a** Event sequence in the role-reversal choice task. A single trial example is shown in which a recorded monkey (M1) was the actor and its partner (M2) was the observer (top). The correct target button (B1, B2, or B3) switched every 11–17 trials, and the actor and observer roles alternated every three trials (bottom). Red arrows indicate switch trials. **b** Three partner conditions. In all conditions, the partner’s action was observed from a frontal point of view. In the FO condition, a wooden stick made the choices. **c** Overall performance (percent correct choice). Mean ± s.e.m. [*n* = 44 (RA), 39 (FM), 40 (FO) sessions for monkey A; *n* = 57 (RA), 67 (FM), 67 (FO) sessions for monkey B]. *ρ* and *P* values, Spearman correlation test. Source data are provided as a Source Data file. **d** Performance (percent optimal choice) after partner choice error. Mean ± s.e.m. [*n* = 44 (RA), 39 (FM), 40 (FO) sessions for monkey A; *n* = 57 (RA), 67 (FM), 67 (FO) sessions for monkey B]. *ρ* and *P* values, Spearman correlation test (two-sided). Dotted lines indicate the chance level (33.3%; see Methods). Asterisks indicate significant differences from chance (**P < 0.01, Student’s *t* test, two-sided): monkey A (RA, *t*_43_ = 4.6, *P* = 7.9 × 10^−5^; FM, *t*_38_ = 11, *P* = 7.3 × 10^−14^; FO, *t*_39_ = 6.8, *P* = 7.4 × 10^−8^) and monkey B (RA, *t*_56_ = 9.7, *P* = 1.3 × 10^−11^; FM, *t*_65_ = 5.3, *P* = 2.0 × 10^−6^; FO, *t*_65_ = 8.8, *P* = 1.4 × 10^−12^). Source data are provided as a Source Data file. **e** Performance (percent optimal choice) after partner switch error. Mean ± s.e.m. [*n* = 44 (RA), 39 (FM), 40 (FO) sessions for monkey A; *n* = 57 (RA), 67 (FM), 67 (FO) sessions for monkey B]. *ρ* and *P* values, Spearman correlation test (two-sided). Dotted lines indicate the chance level (66.7%; see Methods). Asterisks indicate significant differences from chance (*P < 0.05, **P < 0.01, Student’s *t* test, two-sided): monkey A (RA, *t*_43_ = 5.6, *P* = 1.1 × 10^−5^; FM, *t*_38_ = 4.0, *P* = 2.6 × 10^−4^; FO, *t*_39_ = 1.1, *P* = 0.30) and monkey B (RA, *t*_56_ = 4.5, *P* = 1.2 × 10^−4^; FM, *t*_65_ = 2.1, *P* = 0.036; FO, *t*_65_ = 0.42, *P* = 0.67). Source data are provided as a Source Data file. See Supplementary Fig. 1c for results of chi-square tests.

Clarifying these unsolved issues is fundamental to understanding the organizing principles of the primate social brain. To achieve this goal, we developed an experimental procedure in which monkeys alternated making choices with three different types of partners: a real monkey or a human experimenter [real agent (RA) condition, Fig. 1b, left)], a filmed monkey replayed on a monitor (FM condition, Fig. 1b, center), and a filmed object replayed on the monitor (FO condition, Fig. 1b, right). The RA condition is biologically more natural than the FM condition, because a partner in the RA condition really exists in front of the recorded monkey, allowing real-time social interactions, as opposed to a filmed partner in the FM condition that appears and disappears on the monitor abruptly every time a trial starts and ends. Likewise, the FM condition is biologically more natural than the FO condition, because the partner is a biological entity (monkey) in the FM condition as opposed to a nonbiological entity (a wooden stick) in the FO condition. Thus, we operationally defined the biological nature of these partners to be highest in the RA condition, intermediate in the FM condition, and lowest in the FO condition.

Under these conditions, we performed simultaneous, multisite neural recordings in the PMv and MPFC. Here, we report that a sizable number of PMv and MPFC neurons changed their activities depending on the biological nature of observed actions; however, only MPFC neurons exhibited a systematic response bias in favor of biological actions at the population level. By applying the Granger causality analysis to simultaneously recorded neural signals, we obtained evidence that information flow from the PMv to the MPFC increased as the biological nature of observed actions increased. Furthermore, we found by using a double viral vector infection technique^37,38^ that the PMv-to-MPFC pathway was causally involved in monitoring observed, but not executed, actions, for determining optimal choices, particularly when the observed actions were performed by real social agents. Interestingly, the observed deficits in social action monitoring resembled peculiar behavioral disorders reported previously in a monkey with an autistic phenotype^13^.

## Results

### Evaluation of ability to monitor partner’s performance

We used two monkeys (*macaca fuscata*, monkeys A and B; both designated as M1) as experimental subjects for behavioral and neural data collections. M1 was trained to perform a role-reversal choice task (Fig. 1a) with partners (designated as M2) with different degrees of biological nature (Fig. 1b). In the RA condition, M1 and M2 alternated the roles of ‘actor’ and ‘observer’ every three trials (Supplementary Movie 1). In each trial, once both agents had pressed their start buttons, three target buttons on the actor’s side were illuminated simultaneously. The actor was then required to choose one of them by a reach. When the actor chose the target associated with a reward, both M1 and M2 received a water reward; however, when the actor’s choice was wrong, neither was rewarded (Fig. 1a, top). The correct target position remained the same during a block of 11–17 trials (nonswitch trials) and was then changed without prior notice (switch trials; red arrows in Fig. 1a, bottom). The task structure in the two filmed conditions was fundamentally the same (Supplementary Movie 2 for a video stimulus in the FO condition). Both M1s performed the task reasonably well, with the overall correct rate being higher than 75% in all the partner conditions (Fig. 1c). Interestingly, the overall performance of monkey A decreased as M2’s biological nature decreased (Fig. 1c, left; *ρ* = −0.40, *P* = 7.8 × 10^−4^, Spearman correlation test).

We examined whether M1 properly monitored M2’s choice information. To this end, we analyzed M1’s performance after M2’s choice ended in no-rewards. In our task, such no-rewards occurred in both nonswitch and switch trials. In nonswitch trials, no-rewards occurred when, despite no clear evidence that a block switch had occurred, M2 chose a button that was not associated with a reward in the current block (‘choice error’; Supplementary Fig. 1a). This no-reward was caused simply by M2’s erroneous choice. The optimal strategy for M1 following M2’s choice error was to keep choosing the same button that had been rewarded in preceding trials (Supplementary Fig. 1a). In switch trials, no-rewards occurred most typically when M2 chose a button that was associated with a reward in the preceding block (Supplementary Fig. 1b). Thus, this no-reward was caused by an unexpected block switch (‘switch error’). In this case, the optimal strategy was to shift to one of two target buttons that were not rewarded in the preceding block (Supplementary Fig. 1b). We found that the percent optimal choice was significantly above chance in both cases (all *P* < 0.05, Student’s *t* test; Fig. 1d, e), except for post-switch-error trials in the FO condition (monkey A, *t*_39_ = 1.1, *P* = 0.30; monkey B, *t*_66_ = 0.42, *P* = 0.67; Student’s *t* test). These findings indicate that M1 monitored M2’s choice information to determine their own choice. Note, again, that the percent optimal choice following M2’s switch error correlated positively with M2’s biological nature (Fig. 1e; monkey A, *ρ* = −0.16, *P* = 0.012; monkey B, *ρ* = −0.17, *P* = 0.038; Spearman correlation test).

### Difference in actor coding and biological preference between two areas

**Fig. 2:**
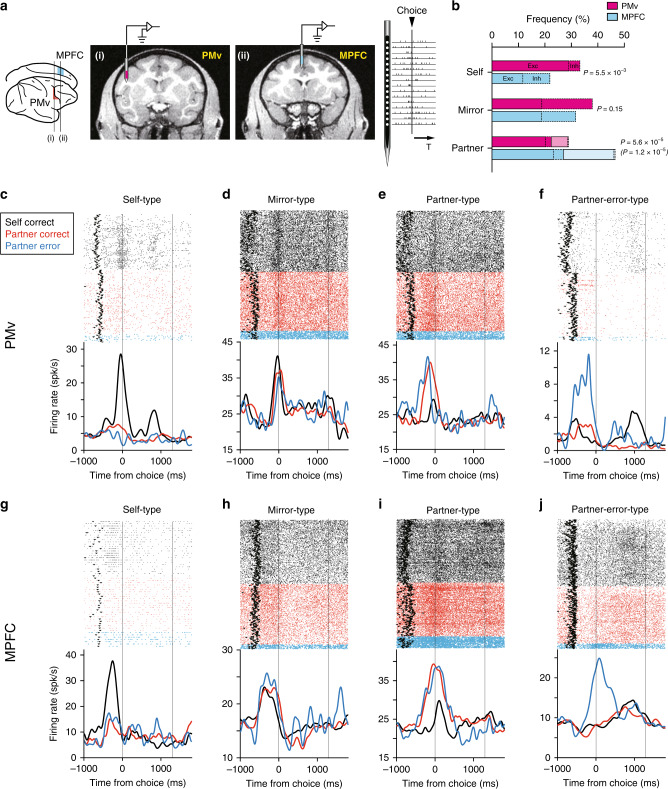
Actor coding in PMv and MPFC. **a** Recording sites of the PMv (i, red) and MPFC (ii, blue). Anteroposterior levels are indicated in the lateral view (left). Single-unit activities were recorded simultaneously in the two areas using two 16-channel electrodes. **b** Proportions of each neuronal type in the PMv (red) and MPFC (blue) in the RA condition. Bars with solid lines, excitatory type; bars with dashed lines; inhibitory type. *P* values, *χ*^2^ test (two-sided). Light-colored bars depict proportions of partner-error-type neurons (*P* value in parenthesis). **c**–**j** Raster displays and spike-density functions aligned to the time of target button press (‘Choice’ in Fig. 1a) for PMv neurons (**c**–**f**) and MPFC neurons (**g**–**j**). Self-type neurons (**c**, **g**), mirror-type neurons (**d**, **h**), partner-type neurons (**e**, **i**), and partner-error-type neurons (**f**, **j**). Colored small dots and black triangles in raster displays indicate the times of individual action potentials and target onset (‘Target on’ in Fig. 1a), respectively. Vertical lines at 1300 ms indicate the time of reward feedback.

We isolated 565 PMv and 480 MPFC single neurons by multisite recordings with no sampling bias (Fig. 2a). Among these, 277 PMv and 215 MPFC neurons exhibited significantly changed activity in the 600-ms period starting 400 ms before the target button press (peri-action period; see Methods). Because direct comparisons of neural activities have never been reported between the PMv and MPFC in the same monkeys performing the same tasks, we first examined actor coding in the RA condition. Most interesting in this respect was the existence of three types of neuronal populations referred to as self, mirror, and partner types (see Methods for the statistical definition of each neuronal type). These populations were found in both areas but with different frequencies (Fig. 2b).

Self-type neurons responded preferentially to the self-action compared to the partner-action (Fig. 2c, g and Supplementary Fig. 2a). This type was significantly more prevalent in the PMv than in the MPFC (PMv, *n* = 92, 33%; MPFC, *n* = 47, 22%; df = 1, *χ*^2^ = 7.7, *P* = 5.5 × 10^−3^, *χ*^2^ test). The response was inhibitory in a subset of neurons (PMv, *n* = 12; MPFC, *n* = 22; Fig. 2b and Supplementary Fig. 3a). Mirror-type neurons responded nondifferentially to the self-action and partner-action (Fig. 2d, h and Supplementary Fig. 2b). The proportion of this type did not differ between the two areas, albeit were more numerous in the PMv than in the MPFC (PMv, *n* = 105, 38%; MPFC, *n* = 68, 32%; df = 1, *χ*^2^ = 2.1, *P* = 0.15). About half of them were negatively modulated (PMv, *n* = 53; MPFC, *n* = 28; Fig. 2b and Supplementary Fig. 3b). Finally, partner-type neurons responded preferentially to the partner-action compared to the self-action (Fig. 2e, i and Supplementary Fig. 2c). In contrast to the self-type, partner-type neurons were significantly more frequent in the MPFC than in the PMv (PMv, *n* = 80, 29%; MPFC, *n* = 100, 47%; df = 1, *χ*^2^ = 16.2, *P* = 5.6 × 10^−5^) and exhibited inhibitory modulations only occasionally (PMv, *n* = 6; MPFC, *n* = 8; Fig. 2b). Partner-type neurons were the most prevalent population in the MPFC (Fig. 2b). The activity of a subset of partner-type neurons was significantly higher during the partner’s incorrect than correct actions (Fig. 2f, j and Supplementary Fig. 2d). This subtype, referred to as the partner-error-type^39^, was identified in both areas but was significantly more common in the MPFC than in the PMv (Fig. 2b; PMv, *n* = 18, 7%; MPFC, *n* = 42, 20%; df = 1, *χ*^2^ = 19.2, *P* = 1.2 × 10^−5^). Across the three types of neurons, response selectivity for the self-action and partner-action varied along a continuum between the two extremes of being almost purely self-selective and partner-selective (Supplementary Fig. 4). Population-averaged activity for each neuronal type aligned with start button onset (i.e., trial start) revealed that such actor selectivity emerged after target onset (Supplementary Fig. 5).

**Fig. 3:**
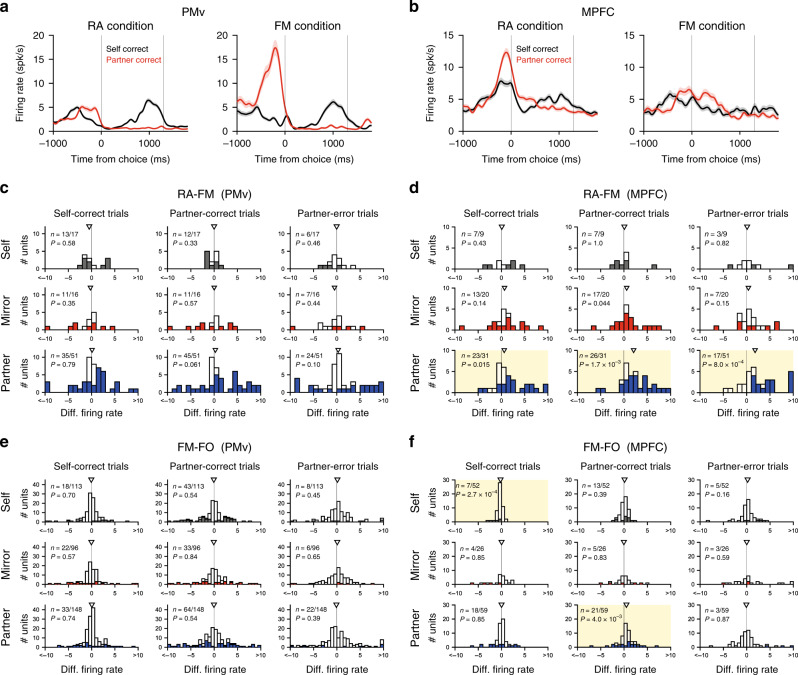
Biological preference in PMv and MPFC. **a** Spike-density functions of a partner-type PMv neuron. Same conventions as in Fig. 2c. Shaded areas indicate s.e.m. **b** Spike-density functions of a partner-type MPFC neuron. **c** Histograms showing differential firing rates, defined as [(activity in the RA condition) − (activity in the FM condition)], for PMv neurons. Self- (black), mirror- (red), and partner-type (blue) neurons (from top to bottom) in self-correct, partner-correct, and partner-error trials (from left to right). Triangles denote the medians. Filled bars indicate neurons with significant activity differences between the two conditions (*P* < 0.01, permutation test, two-sided; see Methods). Fraction numbers in individual histograms denote the number of neurons with significant activity differences relative to the total number of neurons. *P* values, Wilcoxon signed-rank test (two-sided). **d** Differential firing rates between the RA and FM conditions for MPFC neurons. Yellow backgrounds indicate that median values (triangles) are significantly different from zero (*P* < 0.05/3; Wilcoxon signed-rank test with Bonferroni correction, two-sided). Differential firing rates (FM–FO) for PMv neurons (**e**) and MPFC neurons (**f**). Same conventions as in (**c**) and (**d**).

We next examined neuronal preference for specific partner types by comparing the peri-action-period activities during the RA and FM conditions. We found that in both areas the activity of many neurons differed significantly between the two conditions, with some showing a preference for the RA condition and others for the FM condition (Fig. 3a, b). This bidirectional bias was observed in all types at the single-neuron level (Fig. 3c, d). An analysis of covariance using partner’s response time as a covariate revealed that the difference in this kinematic movement parameter did not account for the observed difference in neuronal activity for the great majority of neurons (partner-correct trials, 94%; partner-error trials, 88%). When analyzed at the population level, however, only MPFC neurons exhibited systematic bias in favor of the RA condition (Fig. 3d, yellow background). Specifically, activities of partner-type neurons in the MPFC were significantly greater in the RA condition than in the FM condition during self-correct actions (*z* = 2.4, *P* = 0.015, Wilcoxon signed-rank test), partner-correct actions (*z* = 3.1, *P* = 1.7 × 10^−3^), and partner-incorrect actions (*z* = 3.4, *P* = 8.0 × 10^−4^). Qualitatively similar results were obtained when the partner type was tested separately for the partner-error and non-partner-error types (Supplementary Fig. 6a, b).

A comparison between the FM and FO conditions revealed similar findings. First, the activities of many neurons in both areas changed depending on these two conditions (Fig. 3e, f). Second, at the population level, systematic bias was observed only in the MPFC: activities of partner-type neurons were significantly larger in the FM condition than in the FO condition during partner-correct actions (Fig. 3f; *z* = 3.5, *P* = 4.0 × 10^−3^, Wilcoxon signed-rank test; see also Supplementary Fig. 6c, d). Thus, the results from the two comparisons indicate that both PMv and MPFC neurons are sensitive to the partner’s biological nature on a cell-by-cell basis, but basically only MPFC partner-type neurons show a preferential bias toward the partner of higher biological nature at the population level. Note, however, that a time course analysis using a population-averaged spike-density function revealed that mirror-type and partner-type PMv neurons also exhibited significantly higher activity around the time of target choice in the RA condition than in the FM condition, albeit for a short period of time (Supplementary Fig. 7). The activity of self-type MPFC neurons during the self-action was significantly larger in the FO than in the FM condition (*z* = −3.6, *P* = 2.7 × 10^−4^, Wilcoxon signed-rank test; Fig. 3f), suggesting that even self-action-related activity could be influenced by social contexts.

### Inter-areal coordination between two areas

We next asked whether, and if so, how the PMv and MPFC might coordinate during social action monitoring. To this end, local field potentials (LFPs) were simultaneously recorded in the two areas using 16-channel electrodes. Here, we focused on LFPs recorded when the self-action and partner-action were made correctly, because these trial conditions occurred most frequently and equally often, and thus allowed us to make a more reliable estimate of inter-areal coordination.

**Fig. 4:**
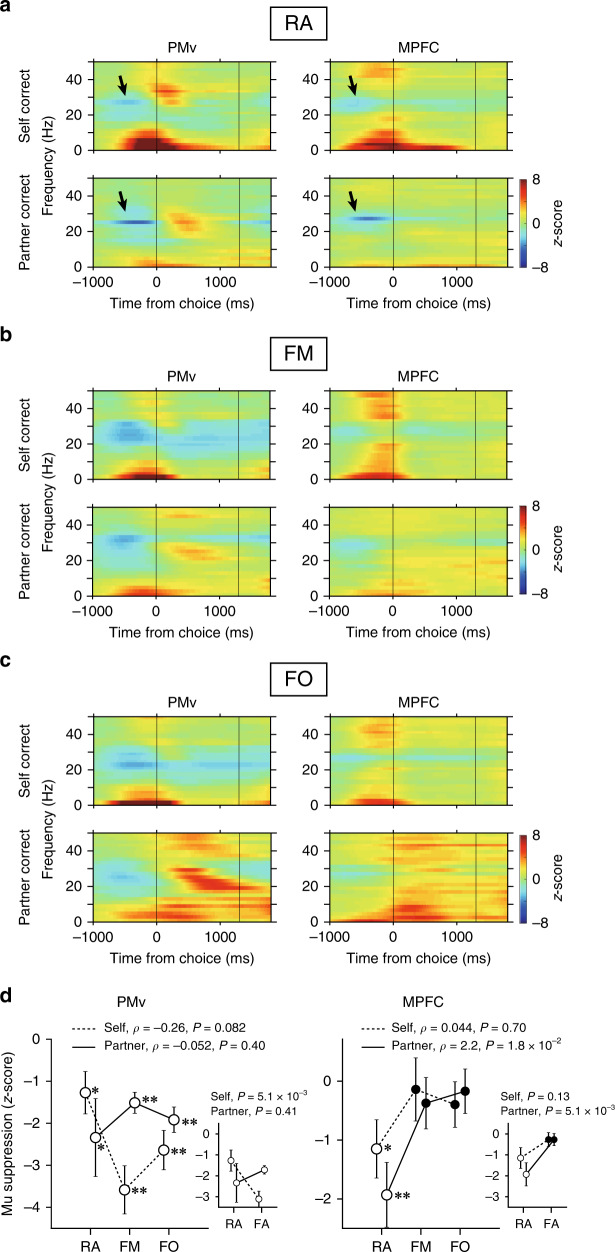
LFP analysis at time-frequency domain in PMv and MPFC. **a**–**c** LFP spectrograms for self-correct trials (top) and partner-correct trials (bottom) [*n* = 28 (RA), 24 (FM), 24 (FO) sessions for PMv; *n* = 30 (RA), 24 (FM), 24 (FO) sessions for MPFC]. Vertical lines at 1300 ms indicate the time of reward feedback. Black arrows in (**a**) indicate mu suppression in the high-beta band. **d** Quantitative analysis of mu suppression (23–30 Hz) in different partner conditions. Mean ± s.e.m. [*n* = 28 (RA), 24 (FM), 24 (FO) sessions for PMv; *n* = 30 (RA), 24 (FM), 24 (FO) sessions for MPFC]. Dotted lines, self-correct trials. Solid lines, partner-correct trials. Open circles indicate that data values are significantly different from zero [*P < 0.05, **P < 0.01; Student’s *t* test, two-sided; PMv self, *P* = 0.020 (RA), 3.1 × 10^−6^ (FM), 1.3 × 10^−5^ (FO); PMv partner, *P* = 0.020 (RA), 5.3 × 10^−5^ (FM), 2.6 × 10^−6^ (FO); MPFC self, *P* = 0.030 (RA), 0.80 (FM), 0.33 (FO); MPFC partner, *P* = 1.8 × 10^−3^ (RA), 0.41 (FM), 0.67 (FO)]. In the MPFC, mu suppression in partner-correct trials positively correlated with partner’s biological nature. *ρ* and *P* values, Spearman correlation test (two-sided). Source data are provided as a Source Data file. In insets, FM and FO are combined and labeled as FA. Mean ± s.e.m. *P* values, Welch’s *t* test (two-sided). Two-way ANOVA for PMv; *P* = 0.74, main effect of actor; *P* = 0.22, main effect of partner; *P* = 0.014, actor × partner interaction. Two-way ANOVA for MPFC; *P* = 0.34, main effect of actor; *P* = 2.3 × 10^−3^, main effect of partner; *P* = 0.34, actor × partner interaction.

We first performed a time-frequency domain analysis. It has been documented that scalp potentials over the human sensorimotor cortex are suppressed in the alpha band during action execution and observation, a phenomenon known as mu suppression^40^. This suppression has been hypothesized to reflect mirror neuron activity^7,40^ and can be weakened or absent in people with ASD^7,41^. In macaques, similar phenomena, but in higher frequency bands, were consistently reported in the PMv^42–45^, whereas possible contributions of the MPFC to mu suppression have not been reported. Here we confirmed in the RA condition that LFP power in the high-beta band (23–30 Hz; black arrows in Fig. 4a, left) was suppressed in the PMv during both self-action and partner-action. Notably, similar suppression also occurred in the MPFC (black arrows in Fig. 4a, right). Critically, the suppression in the MPFC was significant only in the RA condition (Fig. 4a–c, right; asterisks in Fig. 4d, right), which was in marked contrast to the PMv where the suppression was significant in all the partner conditions (Fig. 4a–c, left; asterisks in Fig. 4d, left). In the MPFC, the suppression magnitude for observed actions correlated positively with the biological nature of the partner (Fig. 4d, right; partner-action, *ρ* = 2.2, *P* = 1.8 × 10^−2^; self-action, *ρ* = 0.044, *P* = 0.70; Spearman correlation test). Such systematic modulation was not observed in the PMv either for the self-action (*ρ* = −0.26, *P* = 0.082) or partner-action (*ρ* = −0.052, *P* = 0.40). These findings indicate that mu suppression in the high-beta band co-exists in the PMv and MPFC, but only that for observed actions in the MPFC changes systematically depending on the partner’s biological nature. Interestingly, when the two filmed conditions were combined together [filmed agent (FA) condition], a significant interaction was found between the factor of actors (self vs. partner) and the factor of partner conditions (RA vs. FA) in the PMv [*P* = 0.025, two-way analysis of variance (ANOVA)]. This effect was likely to be caused by enhanced mu suppression in the FA condition during the self-action (*P* = 5.1 × 10^−3^, Welch’s *t* test; Fig. 4d, left, inset).

We also analyzed the dependency of two other LFP components on the partner’s biological nature. First, LFP power below 10 Hz was increased especially prior to the target choice, but this increase was not significantly correlated with the partner’s biological nature in either the PMv (self-action, *ρ* = −0.17, *P* = 0.15; partner-action, *ρ* = −0.15, *P* = 0.21) or the MPFC (self-action, *ρ* = −0.15, *P* = 0.19; partner-action, *ρ* = 0.076, *P* = 0.51; Supplementary Fig. 8). In the PMv, however, the power during the self-action was significantly larger in the RA condition than in the two filmed conditions combined together (i.e., FA condition, as defined above) (*P* = 0.012, actor × partner interaction, two-way ANOVA; *P* = 0.037, post-hoc Welch’s *t* test; Supplementary Fig. 8, inset). Second, LFP power at 18–30 Hz increased after target choice, particularly in the PMv during the partner-action. This increased power was, again, not significantly correlated with the partner’s biological nature (PMv, *ρ* = −0.0034, *P* = 0.98; MPFC, *ρ* = 0.13, *P* = 0.26; Supplementary Fig. 9). However, this LFP component in the MPFC during the partner-action was significantly larger in the FA condition than in the RA condition (*P* = 0.036, actor × partner interaction, two-way ANOVA; *P* = 0.019, post-hoc Welch’s *t* test; Supplementary Fig. 9, right, inset). These findings suggest the existence of frequency-specific, task-phase-dependent processing of social information.

**Fig. 5:**
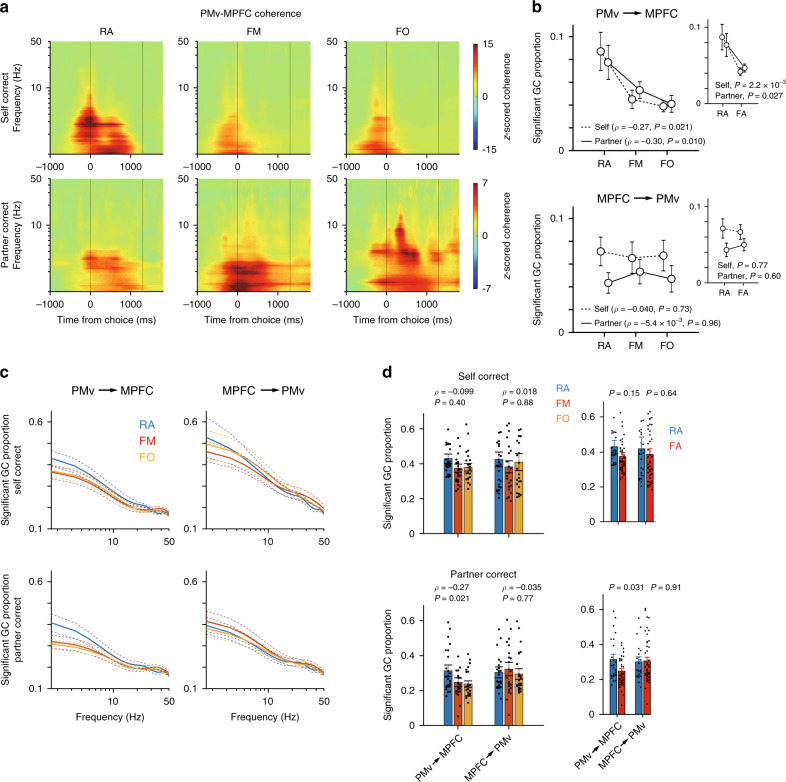
Coherence and Granger causality between PMv and MPFC. **a** Coherent activity between the PMv and MPFC [*n* = 28 (RA), 24 (FM), 24 (FO) sessions]. Top, self-correct trials; bottom, partner-correct trials. Vertical lines at 1300 ms indicate the time of reward feedback. **b** Proportions of channel pairs with significant Granger causality (GC). Median ± s.e.m. [*n* = 27 (RA), 24 (FM), 24 (FO) sessions]. Top, PMv-to-MPFC direction; Bottom, MPFC-to-PMv direction. Dotted lines, self-actions; solid lines, partner-actions. *ρ* and *P* values, Spearman correlation test (two-sided). Source data are provided as a Source Data file. In insets, FM and FO are combined and labeled as FA. Median ± s.e.m. *P* values, Welch’s *t* test (two-sided). Two-way ANOVA for PMv→MPFC; *P* = 0.53, main effect of actor; *P* = 3.7 × 10^−4^, main effect of partner; *P* = 0.71, actor × partner interaction. Two-way ANOVA for MPFC→PMv; *P* = 0.034, main effect of actor; *P* = 0.65, main effect of partner; *P* = 0.63, actor × partner interaction. **c** Significant GC proportions in the frequency domain. Median ± s.e.m. [*n* = 27 (RA), 24 (FM), 24 (FO) sessions]. **d** Comparisons of significant delta-band GC proportions between different partner conditions. Median ± s.e.m. [*n* = 27 (RA), 24 (FM), 24 (FO) sessions]. *ρ* and *P* values, Spearman correlation test (two-sided). Source data are provided as a Source Data file. In insets, FM and FO are combined and labeled as FA. Median ± s.e.m. *P* values, Welch’s *t* test (two-sided). Two-way ANOVA for PMv→MPFC; *P* = 0.028, main effect of actor; *P* = 0.35, main effect of partner; *P* = 0.90, actor × partner interaction. Two-way ANOVA for MPFC→PMv; *P* = 0.97, main effect of actor; *P* = 0.054, main effect of partner; *P* = 0.55, actor × partner interaction.

The above findings raise the possibility that the PMv and MPFC might coordinate in a frequency-specific manner. To test this more directly, we computed field-field coherence between the two areas using the first derivatives of the LFP signals^46^. We found that inter-areal coherence was prominent in the frequency band especially below 3 Hz (Fig. 5a), consistent with the increased low-frequency power in the time-frequency domain analysis (Fig. 4a–c). This coherence increase occurred during both self-action and partner-action in all the partner conditions (Fig. 5a). It is therefore likely that the PMv and MPFC coordinate during social action monitoring mainly via the delta-frequency band. The coherence power in the delta band was not significantly associated with the partner’s biological nature (*ρ* = −6.5 × 10^−3^, *P* = 0.38 for self-correct, *ρ* = 0.086, *P* = 0.49 for partner-correct; Spearman correlation test; Supplementary Fig. 10) or was not significantly different between the RA and FA conditions (self-correct, *P* = 0.37; partner-correct, *P* = 0.52; Welch’s *t* test; Supplementary Fig. 10, inset).

Coherence is silent on the directionality of information flow. Overlapping response latencies between the PMv and MPFC made it difficult to clearly determine which one of the two areas might affect the other (Supplementary Fig. 11). To clarify this point and potential dependence of information flow on the partner’s biological nature, we performed a multivariate Granger causality analysis using the first derivatives of LFPs. We found that Granger causality from the PMv to the MPFC (Fig. 5b, top), but not from the MPFC to the PMv (Fig. 5b, bottom), was greater when the partner’s biological nature was higher for both executed and observed actions (PMv-to-MPFC, *ρ* = −0.27, *P* = 0.021 for self-correct, *ρ* = −0.30, *P* = 0.010 for partner-correct; MPFC-to-PMv, *ρ* = −0.040, *P* = 0.73 for self-correct; *ρ* = −0.0054, *P* = 0.96 for partner-correct; Spearman correlation test). Furthermore, consistent with increased coherence, Granger causality was most pronounced in the delta band (Fig. 5c). In this frequency band, Granger causality in the PMv-to-MPFC direction also correlated positively with biological nature during the partner-action (*ρ* = −0.27, *P* = 0.021; Spearman correlation test; Fig. 5d, bottom-left). These findings suggest that the pathway from the PMv to the MPFC carries signals for social action monitoring, particularly when interacting partners are real animate beings.

### Causal role for PMv-to-MPFC pathway in social action monitoring

**Fig. 6:**
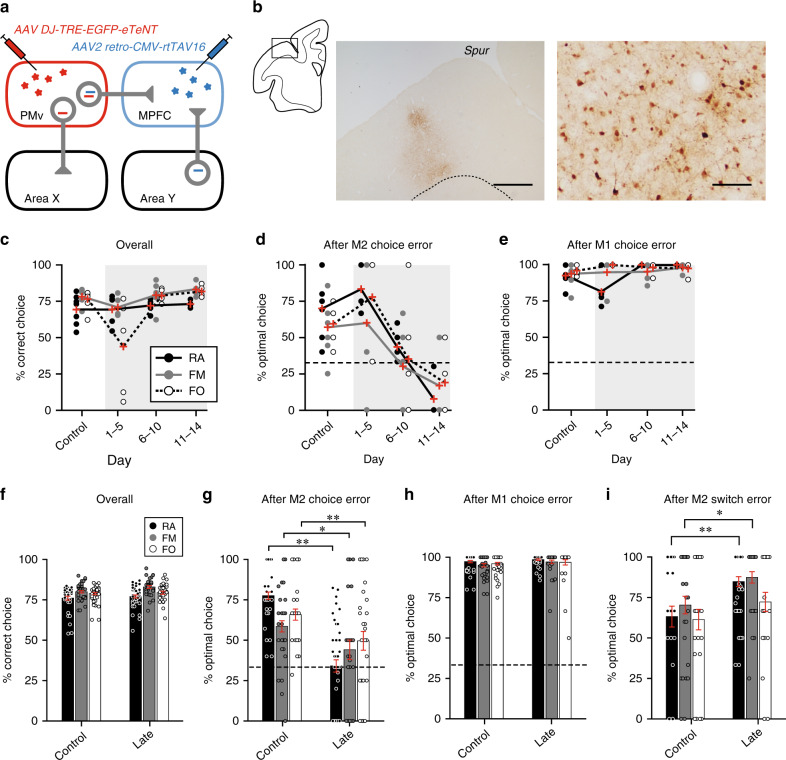
Task performance after selective blockade of PMv-to-MPFC pathway. **a** Pathway-selective blockade using double viral vector technique. Only MPFC-projecting neurons in the PMv are double infected (see Methods). **b** Photomicrographs of a representative injection site in the PMv (*n* = 1 animal). Left, distribution of GFP-positive cells, taken from the rectangular area in the coronal section on the left. Dotted line, gray/white matter border. Scale bar, 2 mm. Right, anti-GFP immunostained cells. Scale bar, 250 µm. Spur, spur of the arcuate sulcus. **c**–**e** Performance before (control) and during (shaded areas) the first Dox administration experiment. Overall percent correct choice (**c**), percent optimal choice after M2’s choice error (**d**), percent optimal choice after M1’s own choice error (**e**). Source data are provided as a Source Data file. Small circles denote data obtained in individual daily sessions. Red crosses denote data averaged across days indicated on the abscissa for each condition. **f**–**i** Performance in control and late Dox periods averaged across four administration experiments. Mean ± s.e.m. [*n* = 28 (RA), 28 (FM), 28 (FO) sessions for the control period; *n* = 38 (RA), 31 (FM), 31 (FO) sessions for the late period]. Overall percent correct choice (**f**), percent optimal choice after M2’s choice error (**g**), percent optimal choice after M1’s own choice error (**h**), percent optimal choice after M2-switch error (**i**). Source data are provided as a Source Data file. *P < 0.05, **P < 0.01, Welch’s *t* test, two-sided (**g**, RA, *t*_64_ = 8.1, *P* = 6.0 × 10^−12^; FM, *t*_57_ = 1.9, *P* = 0.024; FO, *t*_57_ = 2.8, *P* = 7.3 × 10^−3^; **i**, RA, *t*_64_ = −2.9, *P* = 5.3 × 10^−3^; FM, *t*_58_ = −2.5, *P* = 0.014; FO, *t*_58_ = −1.2, *P* = 0.22).

If signals carried by the PMv-to-MPFC pathway are crucial, then selective blockade of this pathway should impair the monitoring of partner’s action. We tested this possibility by employing a viral vector-mediated, pathway-selective intervention in monkey B. In this intervention procedure (Fig. 6a), a retrograde gene transfer vector, AAV2-retro-CMV-rtTAV16, was injected bilaterally into the MPFC (1.1 × 10^13^ viral genomes/ml, 0.25–0.3 μL at 12 sites), while another vector, AAV-DJ-TRE-EGFP-eTeNT-PEST, was injected bilaterally into the PMv (2.1 × 10^13^ viral genomes/ml, 0.25–0.3 μL at 12 sites). Seven weeks later, oral administration of doxycycline (Dox, 0.25–0.3 mg/kg/day) was initiated. With this double viral vector technique^37,38^, PMv neurons whose axons terminate in the MPFC are double infected (Fig. 6a). Dox administration then induces expression of enhanced tetanus neurotoxin (eTeNT), which in turn blocks synaptic transmission of double-infected neurons while other inputs to the MPFC are spared. Using anti-GFP immunohistochemistry, we confirmed that double-infected neurons were localized ventrally to the arcuate spur corresponding to the PMv (Fig. 6b). This PMv sector has been shown to connect abundantly with the MPFC^27,28^.

We performed four courses of Dox administration with an intervening period of at least 4 weeks. In the first experiment, the overall performance was not affected throughout a 14-day test period compared to a 7-day pre-administration control period (Fig. 6c). However, the percent optimal choice decreased gradually, yet steadily and specifically, in trials following partner choice errors (Fig. 6d). This decline in performance was not caused by a failure of no-reward detection or a decrease in general motivation, because the percent optimal choice following M1’s own choice error was not at all affected (Fig. 6e). These observations were consistent across all the Dox experiments: performance was severely impaired after partner choice errors (RA, *t*_64_ = 8.1, *P* = 6.0 × 10^−12^; FM, *t*_57_ = 1.9, *P* = 0.024; FO, *t*_57_ = 2.8, *P* = 7.3 × 10^−3^; Welch’s *t* test; Fig. 6g), whereas neither overall performance (all *P* > 0.05; Fig. 6f) nor performance after M1’s own choice errors was deteriorated (all *P* > 0.05; Fig. 6h). Critically, with the pathway-selective intervention, the performance levels after partner choice error were no longer different from chance level in the RA and FM conditions, while those in the FO condition were still significantly higher than chance (RA, 34%, *t*_65_ = 0.28, *P* = 0.78; FM, 44%, *t*_58_ = 1.7, *P* = 0.10; FO, 50%, *t*_58_ = 2.8, *P* = 8.5 × 10^−3^; Student’s *t* test; Fig. 6g). Furthermore, the performance levels after partner choice error were negatively correlated with the partner’s biological nature after the blockade of the PMv-to-MPFC pathway (*ρ* = 0.19, *P* = 0.044; Spearman correlation test; Fig. 6g). These findings indicate that the PMv-to-MPFC pathway plays a causal role in the monitoring of observed, but not executed, actions for determining one’s own optimal choices. The gaze duration at the partner’s target position was not affected by the intervention (RA, *t*_45_ = −1.0, *P* = 0.31; FM, *t*_45_ = −0.76, *P* = 0.50; FO, *t*_45_ = 1.8, *P* = 0.085; Welch’s *t* test; Supplementary Fig. 12), which ruled out the possibility that the observed impairment was merely due to a loss of overt attention to the partner-action. Interestingly, as opposed to the decline in performance after partner choice errors, the performance levels after partner switch errors were significantly increased in the RA and FM conditions during intervention (RA, *t*_64_ = −2.9, *P* = 5.3 × 10^−3^; FM, *t*_58_ = −2.5, *P* = 0.014; FO, *t*_58_ = −1.2, *P* = 0.22; Welch’s *t* test; Fig. 6i). These opposing intervention effects could be accounted for by deficits in monitoring the partner-action (see Discussion).

## Discussion

In the present study, we directly compared neural activities between the PMv and MPFC in the same monkeys performing the same task, as well as to determine the way in which the two areas might coordinate. We have shown that mirror-type neurons and partner-type neurons are the prevalent population in the PMv and MPFC, respectively, although all three types of actor-coding exist in both areas. We have also shown that, although the classification of neuronal types was made on the basis of statistical criteria, the selectivity for the self-action and partner-action in individual neurons varied along a continuum (Supplementary Fig. 4), like reports in other functional domains, such as visual^47^, oculomotor^48^, and reward^49^ processing. While the continuous distribution of self-coding and partner-coding raises the possibility that the exact boundaries between the three classes of neurons may be somewhat arbitrary, it might also reflect a perceived overlap between the self and others that can be dynamically changed depending on social contexts^50,51^.

We found that at the population level, only partner-type neurons in the MPFC, but not mirror-type or partner-type neurons in the PMv, exhibited a significantly increased response as the biological nature of the partner’s action was increased. The biological preference in the PMv has long been a debate due to mixed evidence in both macaque single-neuron studies^4,14,15,18^ and human neuroimaging studies^16^. Although the activities of a sizable number of PMv neurons did change depending on the partner’s biological nature, our findings suggest that PMv neurons, including the mirror-type, do not exhibit systematic bias in favor of biologically performed actions. Instead, such consistent biological preference is evident in partner-type neurons in the MFPC. This observation accords with neuroimaging findings that the macaque MPFC becomes active during observation of social, but not physical, interactions^3^. The possibility remains, however, that biological preference in the MPFC is dependent, at least partly, on the inputs from the PMv, considering the existence of PMv neurons sensitive to the partner’s biological nature at the single-cell level (Fig. 3c, e), a brief but significant enhancement of PMv activity in the RA condition (Supplementary Fig. 7a), and the partner-dependent information flow from the PMv to the MPFC (Fig. 5b, d).

The time-frequency domain analysis of the LFP signals further supports our conclusion that the PMv and MPFC have different biological preferences. We confirmed that the magnitude of mu suppression in the MPFC, but not in the PMv, changed systematically depending on the biological nature of the partner (Fig. 4d). Mu suppression has been thought to be a neural signature of mirror neuron activity in human studies^7,40^. Our findings now suggest that activity in the MPFC, a midline cortical structure, also contributes to mu suppression in humans. In support of this view, it has been documented that mu suppression can be recorded at Cz, in addition to C3 and C4, in the international 10–20 method of electrode placement^7^. Moreover, the magnitude of mu suppression decreases when observing nonbiological movements compared to biological actions^7^, as found in MPFC LFPs in the present study. These findings suggest that electroencephalogram-based mu suppression contains MPFC-derived response components to a significant degree. The functional significance and clinical relevance of mu suppression in each area are unclear. While the mu frequency band seems to carry information regarding social actions in each of the PMv and MPFC, interactions between the two areas, as indexed by coherent activity, take place in much lower frequency bands (<3 Hz). It has been proposed that slow oscillations serve to synchronize neural activities in large-scale networks^52,53^. These results indicate that social action information is processed in specific frequency channels within (mu frequency band) and between (delta-frequency band) the PMv and MPFC.

We have shown that the distributions of response latencies in the PMv and MPFC overlap substantially (Supplementary Fig. 11). Given a conduction time as fast as a few milliseconds between frontal cortical areas^54,55^, it is still possible that one of the two areas leads the other despite a substantial latency overlap, or separate neural signals are conveyed concurrently in both directions. The Granger causality analysis applied to simultaneously recorded LFPs has revealed the existence of information flow in the PMv-to-MPFC direction that is increased as the partner’s biological nature is increased. Determining what social information might be conveyed in the opposite direction, i.e., from the MPFC to the PMv, is an important future direction.

Consistent with the change in information flow depending on the partner’s biological nature, selective blockade of the PMv-to-MPFC pathway impaired performance after partner choice errors particularly in the RA condition. This impairment cannot be explained by failures to detect no-rewards, lack of attention to other-actions, or decreases in motivation to work. Moreover, further analysis revealed that performance after partner switch errors was significantly better in the RA and FM conditions after intervention (Fig. 6i). This is surprising at first sight, because optimal performance after partner switch errors also requires monitoring other-actions (Supplementary Fig. 1b). How can this apparent puzzle be solved? We hypothesize that blockade of the PMv-to-MPFC pathway causes selective impairments of other-action monitoring, thereby leading to a difficulty extracting task-relevant information from observed actions. We consider that when information about others’ actions becomes virtually unavailable, the monkeys would take a choice strategy that is optimal in the nonsocial condition, that is, action selection based on self-action monitoring and reward feedback, like in the performance of a reversal-learning task in a solo condition. This choice strategy has been described as a ‘win-stay, lose-switch’ (continuation of the same response as long as it is rewarded but changes of the response once it is not rewarded). Under this condition, the monkeys would switch the target whenever no-rewards happens in the partner’s turns regardless of the actual causes of no-rewards (choice error or block switch; Supplementary Fig. 13). Then, choice performance after partner switch errors would be unaffected, or could become even better, because switching of the target is optimal in this case (Supplementary Fig. 1b). By contrast, lower performance levels would be expected after partner choice errors, because switching of the target is nonoptimal in this case (Supplementary Fig. 1a). These are exactly what we observed when the PMv-to-MPFC pathway was selectively blocked. Notably, this pattern of behavioral deficits closely resembles that observed in an autistic monkey with mutations in genes (*HTR2C* and *ABCA13*) linked to human psychiatric and neurodevelopmental disorders^13^. Specifically, the monkey with a spontaneous autistic phenotype in that study exhibited severe performance deficits after partner choice errors, but maintained high performance levels after partner switch errors, during a similar role-reversal choice task. Remarkably, partner-type neurons and mirror-type neurons were almost nonexistent in the MPFC of the autistic monkey^13^.

One of the major questions in social neuroscience is whether, and if so, how the PMv and MPFC coordinate with one another for processing social actions. While a meta-analysis of human neuroimaging has shown that the two areas are rarely concurrently active during social task performance^1^, leading to the proposal that their roles in social cognition are mutually independent or even divergent^21,22^, there have been alternative views that the MPFC may receive social information from the PMv to mediate social cognition^30,35,56^. The present findings indicate that the ability of the MPFC to monitor the other’s action depends on input from the PMv, supporting a coordinative relationship between the two areas. Our findings can also extend the so-called broken mirror hypothesis, which postulates that people with ASD have dysfunctional mirror neurons in the premotor cortex^6–8^. We now suggest that, rather than a deficit in single-neuron types in single brain regions, discoordination between the PMv and the MPFC underlies maladaptive social information processing in ASD. Our intervention procedure selectively targeting the PMv-to-MPFC pathway may provide a useful tool for making a reversible, nonhuman primate model of ASD.

## Methods

### Animals

Two male macaque monkeys [*Macaca fuscata*, monkey A (age 6, 5.1 kg), monkey B (age 6, 5.0 kg)] were used as experimental animals for neural recordings. Other monkeys C (age 8, 8.1 kg) and Q (age 5, 6.3 kg) also participated in this study solely as non-recorded subjects. All animal care and experimentation protocols were approved by the Institutional Animal Care and Use Committee of National Institutes of Natural Sciences and were conducted in accordance with the guidelines described in the US National Institutes of Health Guide for the Care and Use of Laboratory Animals.

### Behavioral procedures

Role-reversal choice task [real agent (RA condition)]: The monkeys were trained to perform the role-reversal choice task (Fig. 1a) with an RA partner (Fig. 1b, left). During data collection, monkey A was paired with monkey B or monkey C, while monkey B was paired with monkey A, monkey C, monkey Q, or a human experimenter. Hereafter, the recorded monkey will be referred to as M1 (or self) and a non-recorded partner will be referred to as M2. In each experimental session, M1 and M2 sat in individual primate chairs facing each other. A square panel was attached horizontally to the front of each chair. Four buttons were placed on the panel: a circular one on the near side toward each participant, which served as a start button, and three rectangular ones on the far side, which served as target buttons. The chair panels for M1 and M2 were closely positioned (distance = ~1 cm; Fig. 1a), such that their target buttons looked continuous from the subjects’ viewpoints.

In each trial, one participant was assigned as the actor and the other the observer. These roles alternated every three trials. Each trial started when the start buttons of both participants turned on. After the participants pressed the start buttons with their right hands for 0.7–1.3 s, the target buttons on the actor’s side turned on. The actor was required to choose one of the three buttons within 3 s, only one of which was associated with a reward. The position-reward association remained the same for a block of 11–17 trials and then was changed unpredictably. A high-pitched tone (1 kHz) occurred together with each target button press regardless of its correctness. The observer was required to hold its start button throughout the trial. When the actor made a correct choice, both participants were rewarded with a drop of water 1300 ms after the target button press. Neither participant was rewarded when the actor made a wrong choice.

Role-reversal choice task [filmed monkey (FM) and filmed object (FO) conditions]: Two filmed conditions were also introduced. In these conditions, a large LCD monitor (W67.41 × H99.56 cm) was placed at the far end of M1’s chair panel; under this setting, the target buttons for M1 and those in the monitor were aligned in the same direction and looked continuous from M1’s viewpoint. In the FM condition, the partner replayed on the monitor was a monkey sitting in a primate chair. In the FO condition, the partner replayed on the monitor was a wooden stick. When the filmed partner was the actor, the monkey or stick on the monitor reached and pressed one of the three targets. When the filmed partner was the observer, the monkey or stick on the monitor kept pressing the start button. A blank screen was presented during intertrial intervals. During the whole experimental period, the overall correct rates for the performance of the filmed partners were set comparable to those of the RA partner (RA, 80%; FM, 78%; FO, 77%; *P* = 0.31, one-way analysis of variance).

To generate visual stimuli for the filmed conditions, actions performed by the filmed partners were recorded beforehand using a video camera (HDR-CX470, Sony, Tokyo, Japan) at 30 frames per second with a resolution of 1920 × 1080 pixels in an uncompressed format. The video sequences were then edited to obtain video clips replayed during actual data collection. The duration and resolution of video clips were 7–8 s and 800 × 860 pixels, respectively. Each video clip started about 1 s prior to the start button onset and ended about 3 s after target button press. Eight to ten different video clips were prepared for each target button choice.

The filmed condition to be tested first in each day was determined randomly, and the two filmed conditions alternated every nine blocks (1 block = 11–17 trials). Where possible, neural recording in the RA condition was also performed before or after the filmed conditions.

### Surgical procedures

A plastic headpost and a plastic chamber were implanted on the skull under aseptic conditions for neuronal recording. The monkeys were initially anesthetized by intramuscular injections of ketamine HCl (10 mg/kg) and xylazine (1–2 mg/kg) and maintained under general anesthesia with isoflurane (1–2%). After the skull was exposed, acrylic screws were installed to fasten a dental acrylic head implant to the skull. A plastic headpost and two recording chambers were placed stereotaxically and secured with dental acrylic. The coordinates of the chambers were determined and confirmed to allow access to the PMv and MPFC bilaterally using magnetic resonance images taken pre- and post-surgery. Antibiotics and analgesics were administered after surgery.

### Behavioral recording procedures

Stimulus presentation, behavioral monitoring, and reward delivery were controlled by a personal computer running the MonkeyLogic Matlab toolbox^57^. Eye position data were streamed to the computer through an infrared video tracking system at a sampling rate of 1 kHz and a spatial resolution of 0.1° (EyeLink II; SR Research, Ontario, Canada). The water reward was delivered through a spout under the control of a solenoid valve placed outside the sound-attenuated room. Overt movements were continuously monitored using a video-capturing system.

### Neuronal recording procedures

Electrophysiological experiments were performed in M1 (monkeys A and B). All sessions for neural recording in the RA condition were performed with a conspecific partner. Single-unit activity and LFPs were recorded simultaneously in the PMv and MPFC using two 16-channel electrodes (U- or S-probe, Plexon Inc., TX, USA), with an interelectrode spacing of 200 μm. The impedance for each channel was 0.3–0.5 MΩ at 1 KHz. Signals were initially amplified and bandpass-filtered (150 Hz to 8 kHz; OmniPlex system; Plexon Inc., TX, USA), and then single-unit activity was isolated using an online template-matching spike discriminator (SortClient; Plexon Inc., TX, USA). All well-isolated neurons were sampled. An oil-driven micromanipulator (MO-97A or MO-971A; Narishige, Tokyo, Japan) was used to advance each probe through a stainless-steel guide tube that was held in place by a grid. The grid allowed recordings every 0.5 mm between penetrations. LFP signals were bandpass-filtered (0.2–500 Hz) and digitized at 1 kHz (OmniPlex system; Plexon Inc., TX, USA) for offline analysis.

### Identification of recording sites

*PMv*: The rostral bank of the arcuate sulcus was initially explored using intracortical microstimulation (ICMS; cathodal pulses of 0.2-ms duration at 333 Hz) to identify the posterior end of the frontal eye field (FEF). Briefly, any penetration was considered to be in the FEF if saccades were evoked by ICMS with low thresholds (typically 11 pulses at a current intensity of <50 μA)^48,58^. During this mapping, the location of the spur of the arcuate sulcus was also noted on the basis of the complete lack of neuronal activity. The premotor cortex was located just caudal to the FEF, and the PMv was identified based on its location relative to the arcuate spur and on distal movements evoked by ICMS^59,60^. During this functional mapping, we ‘clinically’ examined functional properties of well-isolated neurons^60,61^. Briefly, the monkeys were presented with a food item and allowed to grasp it when at reaching distance. To test mirror properties, an experimenter also performed a series of hand actions in front of the monkeys, such as putting a food item on a surface and grasping it.

*MPFC*: The MPFC mainly included the presupplementary motor area (pre-SMA) and its rostrally adjacent prefrontal area 9. We first identified the border between the SMA and pre-SMA according to physiological criteria^54^. Briefly, the face region of the SMA was carefully mapped on the basis of motor effects evoked by ICMS (typically 11–22 pulses at a current intensity of up to 40 μA) and neuronal responses to somatosensory stimuli. The pre-SMA was identified just rostral to the face region in the SMA, and was characterized by (1) a higher threshold for evoking forelimb movements (≥40 pulses and ≥40 μA), (2) complex motor effects involving multiple joints following ICMS, and (3) the prevalence of neurons responsive to visual rather than somatosensory stimuli. The rostral-most portion of the recording site was 12 mm anterior to the border between the SMA and pre-SMA.

### Statistics

No statistical methods were used to predetermine sample sizes, but our sample sizes were similar to those reported in previous publications (for example, see refs. ^37,39,44^). Data were assumed to be normally distributed, but this was not formally tested. We did not select the type of neurons during neural data acquisition; all well-isolated neurons were recorded. Data collection and analysis were not performed blinded to the conditions of the experiments. Correct target positions were pseudorandomly determined. Behavioral analysis was performed using data collected after the initiation of neuronal recordings. No data were excluded, except for LFP time series data with problems of colinearity, nonstationarity, and/or heteroscedasticity for the Granger causality analysis (see below)^62,63^. All the statistical procedures for behavioral and neural data analysis were assessed by two-tailed tests unless otherwise noted and performed in Matlab 2016a and 2017a (MathWorks Inc., Natick, MA, USA) using Statistics and Machine Learning Toolbox, Signal Processing Toolbox, Parallel Computing Toolbox, Control System Toolbox, and Multivariate Granger Causality Toolbox.

### Data analysis

Trial selection and calculation of chance level for M1’s performance following M2’s choice error: To calculate the optimal choice performance (percent optimal choice) following M2’s choice error (e.g., Fig. 1d), we selected M1-actor trials that met the following two conditions: (1) M1-actor trials were immediately preceded by M2’s choice error (i.e., M2 committed a choice error in the third of three consecutive M2-actor trials; Supplementary Fig. 1a, red cross), and (2) the correct target had been revealed, either by the choice of M1 or M2, at least once in the current block before the commission of M2’s choice error. Here, M1’s optimal performance was to choose the correct target in the current block (e.g., if B3 was associated with a reward, then the choice of only B3 was optimal; Supplementary Fig. 1a, gray square). Thus, the percent optimal choice by chance was 33.3%.

Trial selection and calculation of chance level for M1’s performance following M2’s switch error: To calculate the percent optimal choice following M2’s switch error (e.g., Fig. 1e), we selected M1-actor trials that met the following two conditions: (1) M1-actor trials were immediately preceded by M2’s switch error that occurred in the first trial in the current block, and (2) M2’s switch error occurred by choosing the target that was associated with a reward in the preceding block (Supplementary Fig. 1b). In this situation, M1’s optimal performance was to choose one of the two targets that were not associated with a reward in the preceding block (e.g., if B3 was associated with a reward in the preceding block, then the choice of B1 or B2 was optimal; Supplementary Fig. 1b, gray square). Thus, the percent optimal choice by chance was 66.7%.

Neuronal activity: We recorded spike activities of 565 PMv neurons and 480 MPFC neurons. The firing rates of individual neurons were measured during a control period (600–0 ms before target onset) and a peri-action period (from 400 ms before to 200 ms after target button press). We then performed a series of analyses for individual neurons, as follows. First, a two-way analysis of variance (*P* < 0.05) was performed for activity in the peri-action period to test the effects of two factors: agent (self or partner) and performance outcome (correct or incorrect). A neuron was then defined to be agent-selective if it showed a significant main effect of agent and was further classified into one of three types. Specifically, a neuron was classified as a self type (excitatory) if its peri-action-period activity was significantly higher in self-action trials than in partner-action trials (*P* < 0.05, Turkey-Kramer post-hoc test) and, additionally, its peri-action-period activity was significantly higher than the control-period activity in self-action trials (*P* < 0.05, paired *t* test). Alternatively, a neuron was classified as the self type (inhibitory) if its peri-action-period activity was significantly lower in self-action trials than in partner-action trials (*P* < 0.05, Turkey-Kramer post-hoc test) and, additionally, its peri-action-period activity was significantly lower than the control-period activity in self-action trials (*P* < 0.05, paired *t* test).

Likewise, a neuron was classified as a partner type (excitatory) if its peri-action-period activity was significantly higher in partner-action trials than in self-action trials (*P* < 0.05, Turkey-Kramer post-hoc test) and, additionally, its peri-action-period activity was significantly higher than the control-period activity in partner-action trials (*P* < 0.05, paired *t* test). Alternatively, a neuron was classified as the partner type (inhibitory) if its peri-action-period activity was significantly lower in partner-action trials than in self-action trials (*P* < 0.05, Turkey-Kramer post-hoc test) and, additionally, its peri-action-period activity was significantly lower than the control-period activity in partner-action trials (*P* < 0.05, paired *t* test). A partner-type neuron could be further classified as a partner-error type if it also showed a significant main effect of performance outcome. Specifically, an excitatory partner-type neuron was defined as an excitatory partner-error-type neuron if it exhibited peri-action-period activity that was significantly higher in partner-error trials than in partner-correct trials (*P* < 0.05, Turkey-Kramer post-hoc test). Conversely, an inhibitory partner-type neuron was defined as an inhibitory partner-error-type neuron if it exhibited peri-action-period activity that was significantly lower in partner-error trials than in partner-correct trials (*P* < 0.05, Turkey-Kramer post-hoc test). Finally, a neuron was classified as a mirror type if it lacked a significant main effect of agent, but its peri-action-period activity was significantly higher (excitatory) or lower (inhibitory) than the control-period activity in both self-action and partner-action trials (*P* < 0.05, paired *t* test).

In constructing continuous spike-density functions for populations of neurons, each spike was convolved with a Gaussian kernel (SD = 30 ms) for each neuron. The resulting spike densities for individual neurons were normalized from zero to maximum and were averaged for each neuron type.

To examine a biological preference of individual neurons, activity in the peri-action period was compared between the RA and FM conditions, and between the FM and FO conditions (*P* < 0.01, permutation test with 1000 iterations). Also, to examine a biological preference of the population of PMv neurons and MFC neurons, we constructed a histogram illustrating the distribution of differential firing rates. The differential firing rate was defined for each excitatory neuron as [(peri-action-period activity in the RA condition) minus (peri-action-period activity in the FM condition)] for the RA–FM comparison and [(peri-action-period activity in the FM condition) minus (peri-action-period activity in the FO condition)] for the FM–FO comparison. For each inhibitory neuron, the sign of this value was reversed. We then tested whether the differential firing rate at the population level was significantly different from zero (*P* < 0.05/3, Wilcoxon signed-rank test with Bonferroni correction). This comparison was made separately for each neuron type.

To compute actor selectivity for individual neurons (Supplementary Fig. 4b), we calculated another differential firing rate, which was defined as [(peri-action-period activity during partner-action) minus (peri-action-period activity during self-action)]. Here, self-action was defined as the action in self-correct trials. Partner-action was defined as the action in partner-error trials for partner-error-type neurons and the action in partner-correct trials for the other types of neurons. Again, the sign of the value was reversed for each inhibitory neuron.

Time-frequency domain analysis: We calculated time-varying power spectra of LFPs^46^. Briefly, power in each frequency band was computed in 1-ms steps and in 1-Hz steps from 1 to 50 Hz. Each time-varying power spectra was normalized per frequency by the activity 500–0 ms before the target onset using a *z*-score normalization procedure and averaged across sessions. The strength of LFP power was quantified by averaging the *z*-scored time-varying power spectra (1–10 Hz and 23–30 Hz, −600 to 0 ms from target button press; 18–30 Hz, 200–1000 ms from target button press; Fig. 4d and Supplementary Figs. 8 and 9).

Field-field coherence: For each 16-channel electrode in the PMv and MPFC, the first derivative of the LFPs from adjacent channels were computed in the superficial direction to generate 15 bipolar LFPs. This procedure is known to effectively attenuate potential artifacts caused by electric volume conduction and the common reference, resulting in more spatially precise evaluation of signal interactions^46,64^. The procedures for calculating coherence using bipolar LFPs were described in detail elsewhere^62^. Briefly, the bipolar LFPs from 2.5 s before to 2.5 s after target button press were concatenated for each recording session into one long time series for self-correct and partner-correct trials. The concatenated signals were then convolved with a complex Morlet wavelet function and divided into the original 5-s LFP segments. Coherence was calculated for all LFP pairs between the PMv and MPFC ranging from 1 to 50 Hz in a logarithmic step. Each coherence profile was normalized in the same way as the time-varying power spectra and averaged across sessions. To quantify the strength of delta-band coherence, *z*-scored coherent data were averaged between 1 and 3 Hz during a 300-ms period immediately before target button press (Supplementary Fig. 10).

Granger causality: To evaluate the direction of information flow between the PMv and MPFC, a Granger causality analysis^65^ was applied to the bipolar LFPs simultaneously recorded in the two areas. This analysis was implemented using a multivariate linear vector autoregressive (MVAR) model provided by the Multivariate Granger Causality Toolbox^63^ and described in detail elsewhere^66^. Briefly, the bipolar LFP segments (300–0 ms before target button press) were analyzed. Akaike information criteria was used to estimate the best model order up to 50 ms. The MVAR model parameters for the selected model order were estimated using ordinary least-squares regression. The autocovariance sequence from the MVAR parameters was calculated for the LFP time series data without problems of colinearity, nonstationarity, and/or heteroscedasticity. Finally, the time-domain pairwise conditional Granger causality was estimated using F-testing with false discovery rate (*Q* < 0.05). The numbers of channel pairs with significant Granger causality for the PMv-to-MPFC direction and the MPFC-to-PMv direction were counted for quantitative comparisons. The Granger information flow was also estimated in the frequency domain between 1 and 120 Hz with a 1.67-Hz resolution, again using F-testing with false discovery rate (*Q* < 0.05). For quantitative comparisons, the number of channel pairs with significant Granger causality was counted in the delta-frequency band (1–3 Hz) where high coherent activities were observed.

### Pathway-selective intervention

Injection of viral vectors: To selectively block the synaptic transmission of PMv neurons whose axons terminate in the MPFC, the genetic dissection method using two viral vectors^37,38^ was applied to monkey B. A retrograde gene transfer adeno-associated viral vector serotype 2, which carried an enhanced reverse tetracycline trans-activator (AAV2-retro-CMV-rtTAV16, 1.1 × 10^13^ viral genomes/mL), was injected into the MPFC. A different serotype of the AAV vector, AAV-DJ, which carried enhanced tetanus neurotoxin light chain (eTeNT) and enhanced green fluorescent protein (eGFP) downstream of the tetracycline-responsive element (TRE) (AAV-DJ-TRE-EGFP-eTeNT-PEST, 2.1 × 10^13^ viral genomes/mL), was injected into the PMv. With these injections, PMv neurons whose axons terminate in the MPFC were double infected by the vectors (Fig. 6a, b).

Vector injections were made bilaterally by pressure through a 10-μL Hamilton microsyringe. For the MPFC, the AAV2-retro injections were made into three sites centered 6.5 mm rostral from the SMA/pre-SMA border in each hemisphere. The viral vector was deposited at two different depths for each site, aiming at 2.0 and 3.5 mm from the cortical surface (0.25–0.3 μL at each depth). For the PMv, the AAV-DJ injections were made into three sites centered 1.5 mm caudal from the posterior end of the arcuate sulcus and 3 mm ventral from the arcuate spur in each hemisphere. The viral vector was deposited at two different depths for each site aiming at 1.5 and 3.0 mm from the cortical surface (0.25–0.3 μL at each depth). The injection sites in the PMv and MPFC were determined on the basis of the finding that task-related neurons were abundantly found at those sites. Note that these sites in the two areas correspond to cortical locations with known mutual anatomical connections^23,26^.

Dox administration: We performed four courses of Dox administration. The first course was initiated 7 weeks after the vector injections. Dox was administered orally at a dose of 25–30 mg/kg/day. The first to the fourth experiments were continued for 14, 18, 20, and 50 days, respectively, with an interval of at least 4 weeks. Control data were collected for 7 days before Dox administration. Behavioral data during Dox administration were collected every day except the fourth course [*n* = 18 (RA), 7 (FM), and 7 (FO) days for the fourth course]. The Dox test period was divided into an early and a late half for statistical analysis. During the Dox administration experiments, monkey B performed the task with a conspecific or a human experimenter. The overall correct rates were comparable with a monkey partner (80%, *n* = 54 sessions) and with a human partner (78%, *n* = 47 sessions; *P* = 0.22, Welch’s *t* test); data with two RA conditions were thus combined.

Gaze behavior: Possible changes in gaze behavior due to Dox administration were evaluated. Specifically, the proportion of the subject’s gaze at the partner’s correct target button was compared before (control period) and after (late test period) Dox administration (*P* < 0.05, Welch’s *t* test). For this comparison, M1’s gaze positions were quantified during a 300-ms period before the target button press. Eye positions in the fourth course were not available due to a technical error during data collection.

### Histology

After the Dox administration experiments, monkey B was anesthetized deeply with sodium pentobarbital (70 mg/kg, i.v.) and transcardially perfused with 0.1 M phosphate-buffered saline (PBS; pH 7.4) and then 10% formalin in 0.1 M phosphate buffer (pH 7.4). The brain was removed from the skull and postfixed overnight. After saturated with 30% sucrose for 2 weeks at 4 °C, the brain was sectioned coronally at 50 μm thickness. A series of every tenth section was initially treated with 0.3% hydrogen peroxide in 0.1 M PBS for 30 min at room temperature to inhibit endogenous peroxidase. Subsequently, the sections were immersed in 1% skim milk for 1 h and incubated overnight at 4 °C with rabbit anti-GFP antibody (1:3000; Thermo Fisher Scientific, MA, USA) in 0.1 M PBS containing 0.1% Triton X-100 and 1% horse serum. The sections were incubated for 2 h in the same medium containing biotinylated horse anti-rabbit IgG antibody (1:200; Vector Laboratories, Peterborough, UK) and reacted with the ABC Elite kit (Vector Laboratories) for 1.5 h. For visualization of the antigen, the sections were reacted in 0.1 M PBS containing 0.04% 3,3′-diaminobenzidine and 0.002% hydrogen peroxide. An adjacent series of sections was Nissl-stained with 5% Cresyl Violet.

### Reporting summary

Further information on research design is available in the Nature Research Reporting Summary linked to this article.

## Supplementary information

Supplementary Information

Supplementary Movie 1

Supplementary Movie 2

Reporting Summary

## Data Availability

The authors declare that the main data supporting the findings of this study are available within the paper and its supplementary information files. Extra data are available from the corresponding author upon reasonable request. Source data are provided with this paper.

## Source data

Source Data

## Code availability

Analyses were performed by using Matlab toolboxes (see Reporting summary for details). Matlab scripts employed are available from the corresponding author upon reasonable request.
