# Supplementary material for: A causal role for frontal cortico-cortical coordination in social action monitoring
Source: Nat Commun. 2020 Oct 16;11:5233. doi: 10.1038/s41467-020-19026-y (PMC7568569; doi:10.1038/s41467-020-19026-y)
Supplement: Supplementary file 1 — Supplementary Information [file 41467_2020_19026_MOESM1_ESM.pdf]

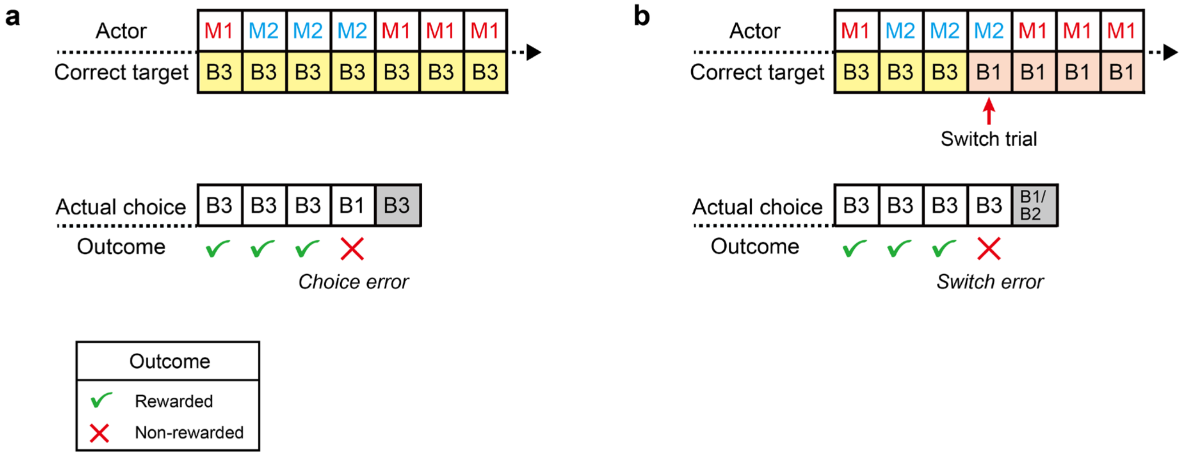

c

Monkey A			
Partner	# switch error	# choice error	χ2 test
RA	556/611 (91)	15/81 (18)	χ2=260, P=1.4×10^{-58}
FM	208/250 (83)	26/111 (23)	χ2=389, P=1.2×10^{-86}
FO	126/182 (69)	30/99 (30)	χ2=39, P=3.6×10^{-10}

Monkey B			
Partner	# switch error	# choice error	χ2 test
RA	262/298 (88)	32/139 (23)	χ2=381, P=7.1×10^{-85}
FM	247/313 (79)	77/193 (40)	χ2=157, P=3.2×10^{-36}
FO	221/351 (63)	59/183 (32)	χ2=22, P=2.6×10^{-6}

Supplementary Fig. 1. Illustration of two types of no-rewards caused by M2's choices. a Choice error made by M2. Choices in white squares denote actual choices by the actor (M1 or M2) and those in gray squares denote M1's optimal choices. Green check marks indicate rewarded outcomes and red crosses indicate non-rewarded outcomes (inset). **b** Switch error made by M2. In this case, a switch error occurred in the third of three consecutive M2-actor trials. Same conventions as in (**a**). See Methods for calculation of chance-level performance. **c** Fraction number denotes the number of switched choices relative to the total number of partner's switch errors or choice errors. Numbers in parentheses denote the percentages. P values, chi-square test (two-sided).

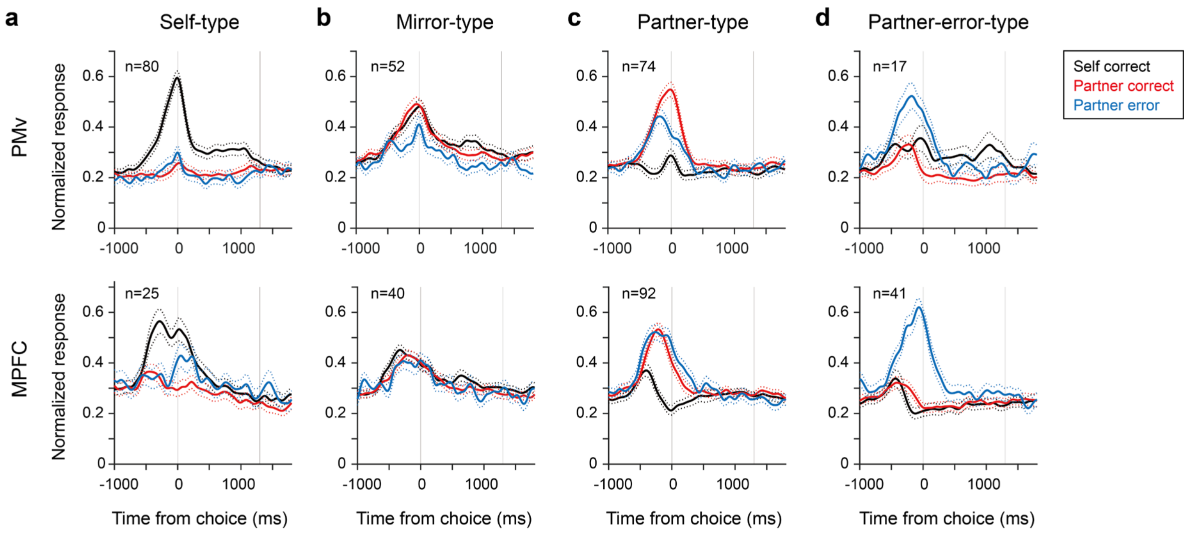

Supplementary Fig. 2. Population activity (excitatory modulation). a–d Population-averaged spike density functions for each neuron type with excitatory modulation in the RA condition. Self-type (**a**), mirror-type (**b**), partner-type (**c**), and partner-error-type (**d**) neurons in the PMv (top) and MPFC (bottom). Continuous and dotted lines represent mean and s.e.m., respectively. Other conventions are as in Fig. 2c.

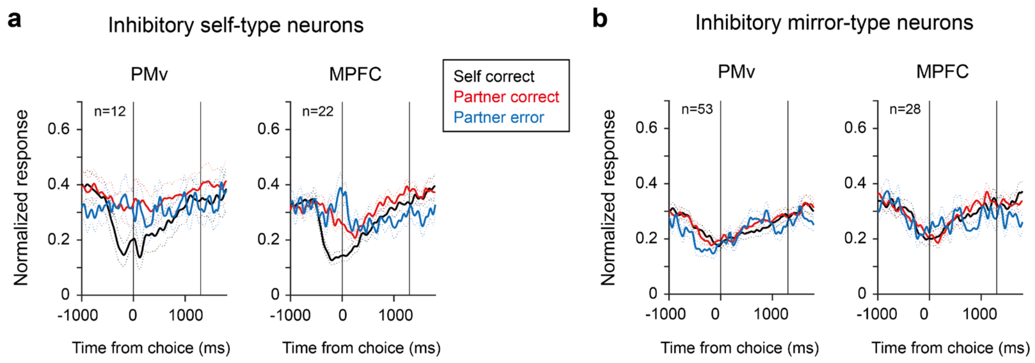

Supplementary Fig. 3. Population activity (inhibitory modulation). a,b Population-averaged spike density functions for self-type (**a**) and mirror-type (**b**) neurons with inhibitory modulation in the RA condition. Other conventions are as in Supplementary Fig. 2.

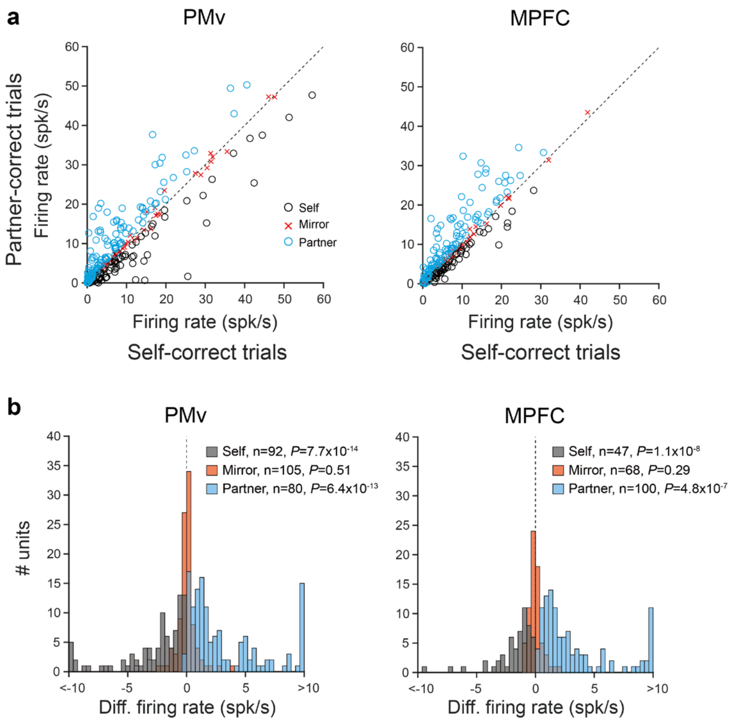

Supplementary Fig. 4. Comparison of actor selectivity between self-type, mirror-type, and partner-type neurons. a Scatter plot of peri-action-period activity during the self-action (abscissa) and partner-action (ordinate) in the RA condition. Results from non-partner-error-type and partner-error-type neurons were combined and labeled collectively as Partner. **b** Differential firing rates, activity during the partner-action minus activity during the self-action. *P* values, Wilcoxon signed-rank test (two-sided).

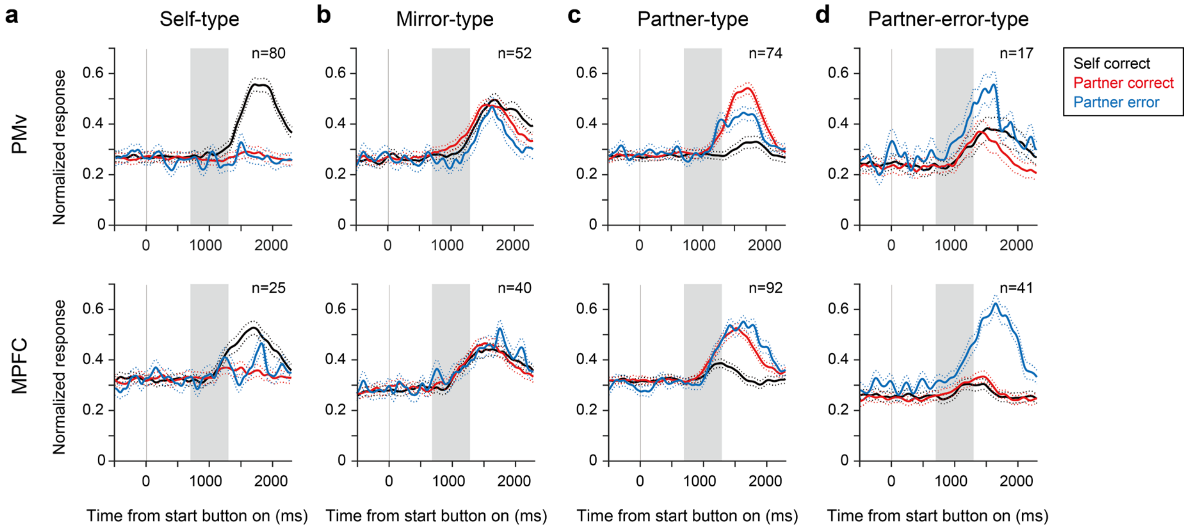

Supplementary Fig. 5. Population activity aligned to the start button onset (excitatory modulation). a–d Population-averaged spike density functions for each neuronal type with excitatory modulation in the RA condition. Continuous and dotted lines represent mean and s.e.m., respectively. The target button turned on 0.7–1.3 s after the start button onset (shaded areas). Other conventions are as in Fig. 2c.

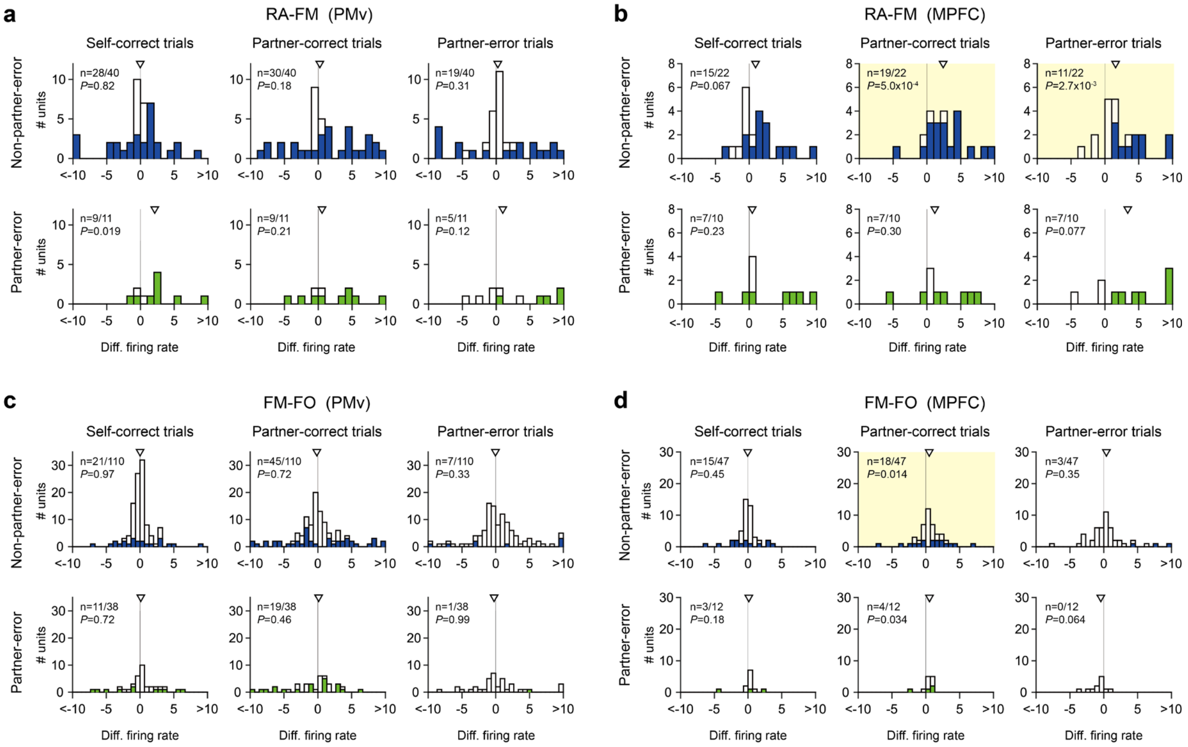

Supplementary Fig. 6. Biological preference in partner-type neurons. a,b Histograms showing differential firing rates between the RA and FM conditions. PMv (**a**) and MPFC (**b**). Partner-error-type and non-partner-error-type neurons are shown separately. Other conventions are as in Fig. 3c,d. **c,d** Histograms showing differential firing rates between the FM and FO conditions. PMv (**c**) and MPFC (**d**). Yellow backgrounds indicate that median values (triangles) are significantly different from zero ($P < 0.05/3$, Wilcoxon signed-rank test with Bonferroni correction, two-sided). Other conventions are as in Fig. 3e,f.

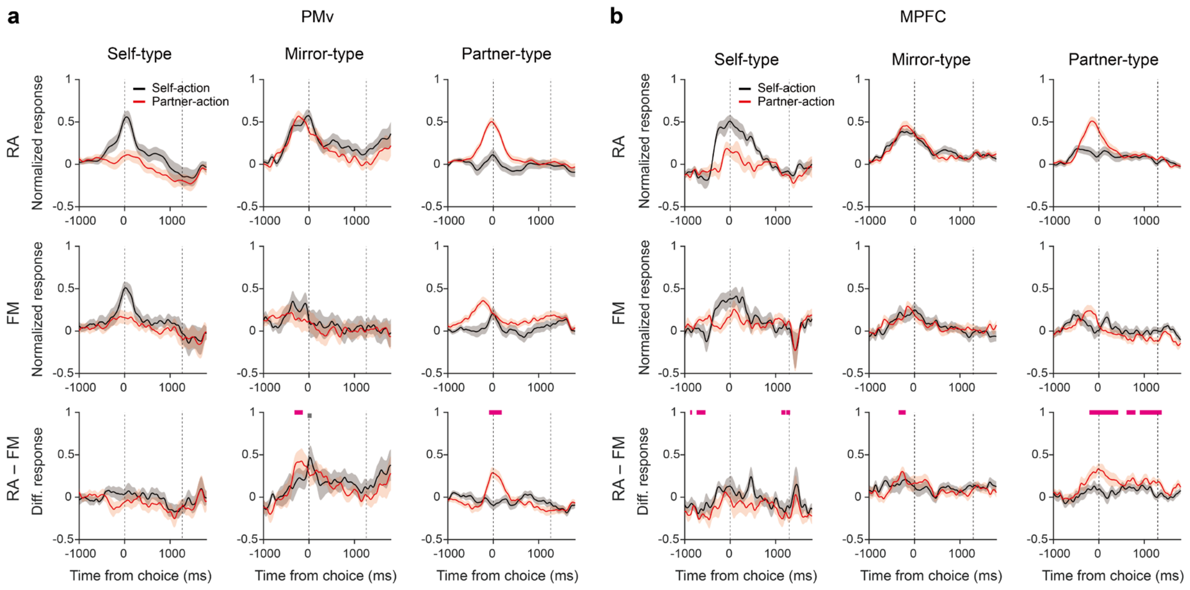

Supplementary Fig. 7. Time courses of activity difference between RA and FM conditions. a,b Population-averaged spike density functions for each neuronal type in the RA condition (top), FM condition (middle), and their difference (bottom) in the PMv (**a**) and MPFC (**b**). These plots were derived from neuronal data used in Fig. 3. Continuous and dotted lines represent mean and s.e.m., respectively. Other conventions are as in Fig. 2c. For differential responses at the bottom, gray (self-action) and magenta (partner-action) bars denote the time at which data points were significantly different from zero ($P < 0.01$, Wilcoxon signed-rank test, two-sided).

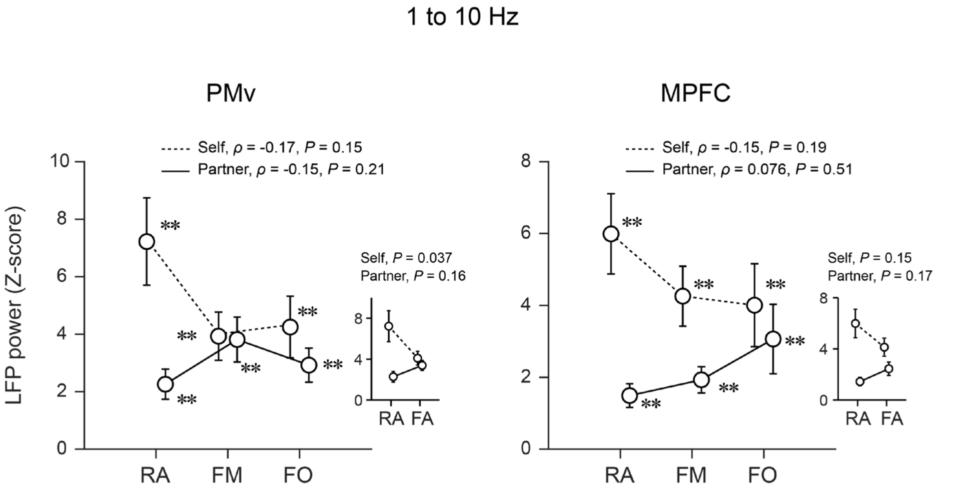

Supplementary Fig. 8. Quantitative analysis of LFP power in low frequency bands. Comparisons of LFP power in 1–10 Hz between different partner conditions. Mean ± s.e.m [n = 28 (RA), 24 (FM), 24 (FO) sessions]. ρ and P values, Spearman correlation test (two-sided). Open circles indicate that data values are significantly different from zero [$*P < 0.05$, $**P < 0.01$; Student's t-test, two-sided; PMv self, $P = 7.9\times10^{-5}$ (RA), 1.3×10^{-4} (FM), 7.7×10^{-4} (FO); PMv partner, $P = 2.2\times10^{-4}$ (RA), 7.9×10^{-5} (FM), 6.6×10^{-5} (FO); MPFC self, $P = 1.3\times10^{-5}$ (RA), 4.7×10^{-5} (FM), 2.5×10^{-3} (FO); MPFC partner, $P = 1.9\times10^{-4}$ (RA), 4.0×10^{-5} (FM), 5.6×10^{-3} (FO)]. In insets, FM and FO are combined and labeled as FA. Mean ± s.e.m. P values, Welch's t-test (two-sided). Two-way ANOVA for PMv; $P = 9.0\times10^{-4}$, main effect of actor; $P = 0.23$, main effect of partner; $P = 0.012$, actor × partner interaction. Two-way ANOVA for MPFC; $P = 3.9\times10^{-5}$, main effect of actor; $P = 0.56$, main effect of partner; $P = 0.053$, actor × partner interaction. Source data are provided as a Source Data file.

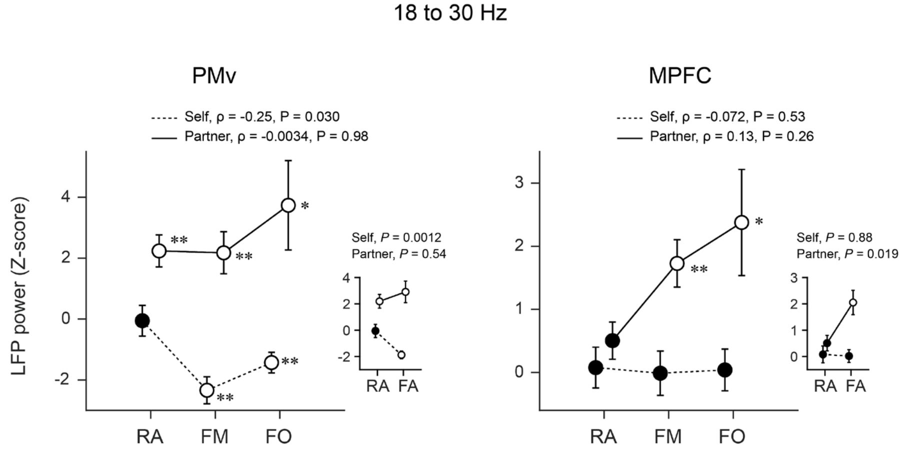

Supplementary Fig. 9. Quantitative analysis of LFP power after choice. Comparisons of LFP power in 18–30 Hz between different partner conditions (200–1000 ms after choice). Mean ± s.e.m [n = 28 (RA), 24 (FM), 24 (FO) sessions]. ρ and P values, Spearman correlation test (two-sided). Open circles indicate that data values are significantly different from zero [$*P < 0.05$, $**P < 0.01$; Student's t-test, two-sided; PMv self, $P = 0.91$ (RA), 3.1×10^{-5} (FM), 3.7×10^{-4} (FO); PMv partner, $P = 3.6\times10^{-4}$ (RA), 6.2×10^{-3} (FM), 0.022 (FO); MPFC self, $P = 0.81$ (RA), 0.97 (FM), 0.90 (FO); MPFC partner, $P = 0.10$ (RA), 1.6×10^{-4} (FM), 0.011 (FO)]. In insets, FM and FO are combined and labeled as FA. Mean ± s.e.m. P values, Welch's t-test (two-sided). Two-way ANOVA for PMv; $P = 2.4\times10^{-7}$, main effect of actor; $P = 0.39$, main effect of partner; $P = 0.052$, actor × partner interaction. Two-way ANOVA for MPFC; $P = 1.5\times10^{-3}$, main effect of actor; $P = 0.053$, main effect of partner; $P = 0.036$, actor × partner interaction. Source data are provided as a Source Data file.

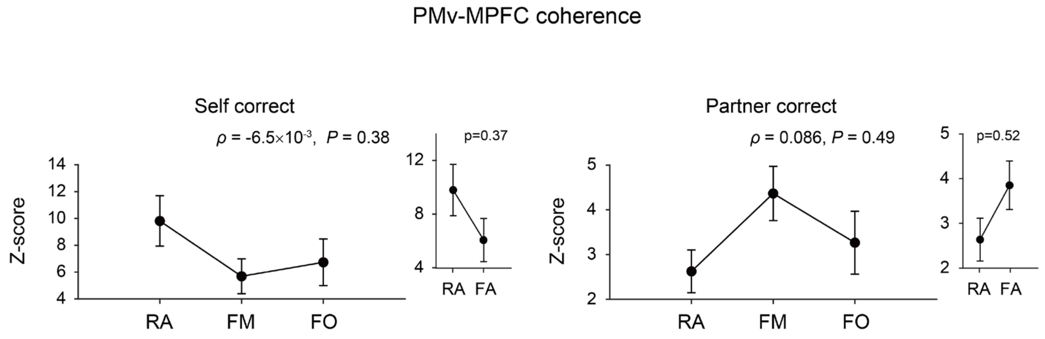

Supplementary Fig. 10. PMv-MPFC coherence. Comparisons of coherence power in the delta band between different partner conditions. Mean ± s.e.m [n = 28 (RA), 24 (FM), 24 (FO) sessions]. ρ and *P* values, Spearman correlation test (two-sided). Insets, FM and FO are combined and labeled as FA. Mean ± s.e.m. *P* values, Welch's *t*-test (two-sided). Source data are provided as a Source Data file.

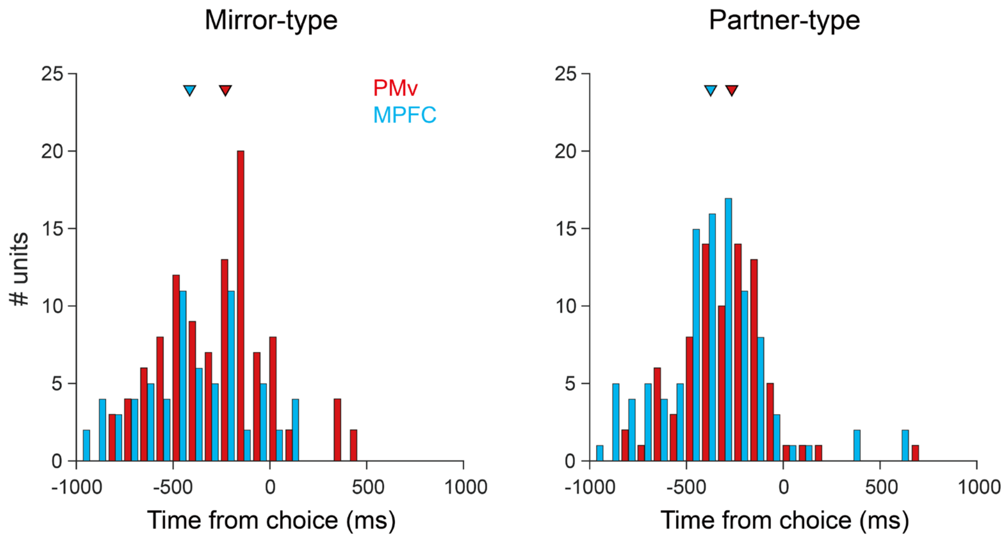

Supplementary Fig. 11. Comparison of response onset (latency) between PMv neurons and MPFC neurons. The distributions of response onset relative to the time of choice for PMv neurons (red) and MPFC neurons (blue) in the RA condition. Self-correct and partner-correct trials, partner-correct trials, and partner-error trials were used to calculate response onset for mirror-type, partner-type, and partner-error-type neurons, respectively. For each neuron, activity during the control period (600–0 ms before target onset) was averaged to estimate the baseline activity. The response onset was then defined as the first bin (1-ms resolution) at which averaged activity aligned to the time of choice exceeded ± 3 s.d. from the baseline continuously for more than 30 bins. Results obtained from partner-type and partner-error-type neurons were combined (right). Triangles denote the median. Mirror-type, n = 105 for PMv and n = 68 for MPFC, $P = 3.7\times10^{-3}$; partner-type, n = 80 for PMv and n = 100 for MPFC, $P = 0.54$; Welch's *t*-test (two-sided).

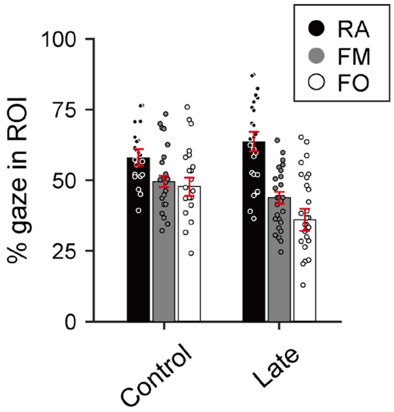

Supplementary Fig. 12. Gaze duration before and after Dox administration. Percent duration of M1's gaze within a region of interest (ROI) set to cover the partner's correct target button. Mean ± s.e.m. (n = 21 sessions for the control period; n = 26 sessions for the late period). Other conventions are as in Fig. 6f. Source data are provided as a Source Data file.

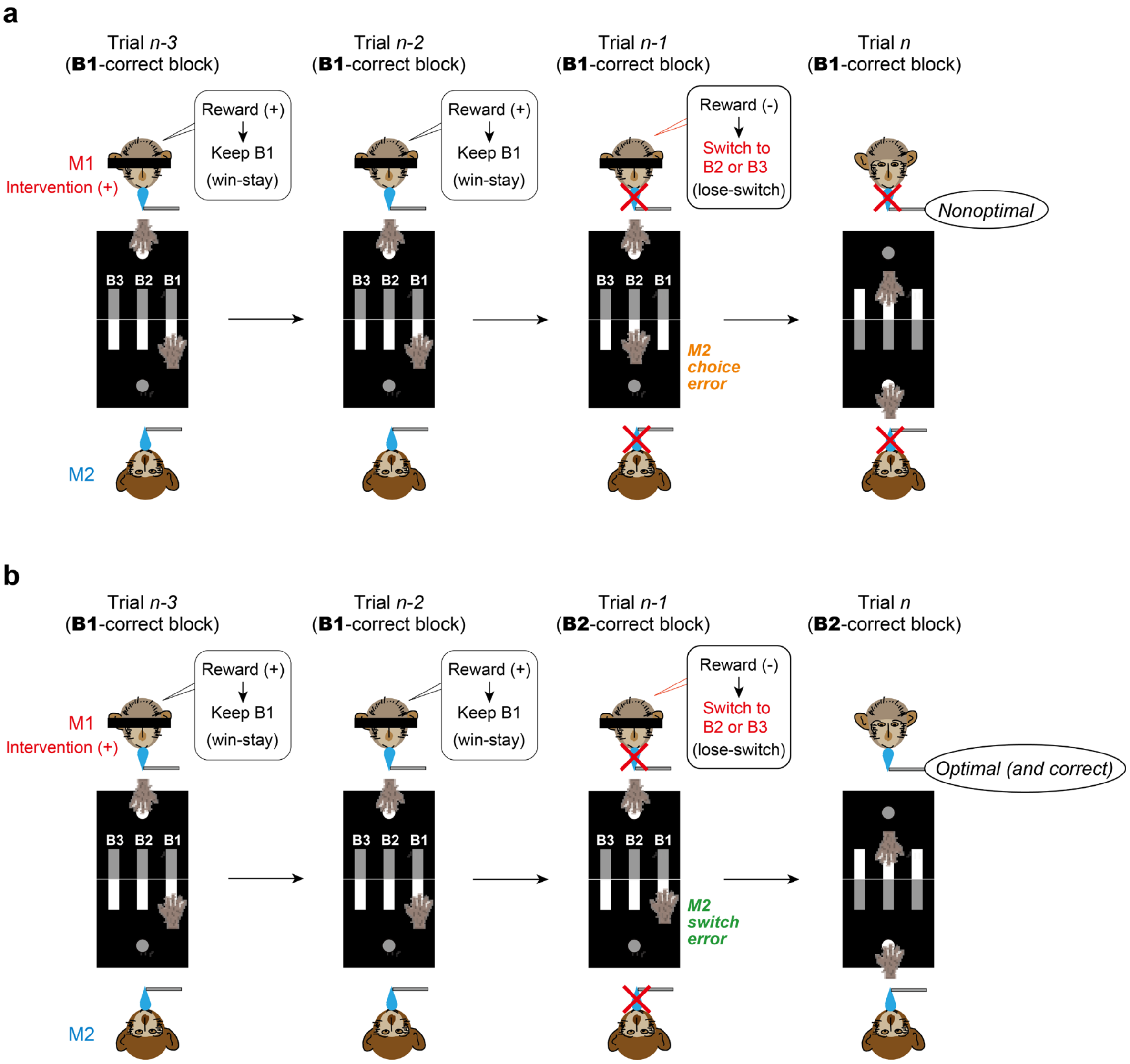

Supplementary Fig. 13. Hypothetical explanation of decreased performance levels after M2 choice error and increased performance levels after M2 switch error during intervention experiments for M1. a,b For illustrative purposes, selective impairment of partner-action monitoring is depicted by M1 with a blinder in M2-actor trials. Note that, during intervention, M1 takes a win-stay, lose-switch strategy on the basis of reward-feedback. **a** Performance after M2 choice error. Consider a series of trials in which B1 is the correct target and nonswitch trials continue. M1 keeps in mind that B1 is the correct target as long as a reward is delivered (trials *n-3* and *n-2*), but switches to a non-B1 target (B2 or B3) once a reward is not delivered (trial *n-1*). M1's selection of B2 or B3 in trial *n* is nonoptimal, because the correct target remains unchanged (i.e., B1). In this manner, the percentage of optimal choices is decreased after M2 choice error (Fig. 6g). **b** Performance after M2

switch error. Consider a series of trials in which the correct target is switched from B1 to B2 in trial *n-1*. Again, M1 keeps in mind that B1 is the correct target as long as a reward is delivered (trials *n-3* and *n-2*), but switches to a non-B1 target (B2 or B3) once a reward is not delivered (trial *n-1*). M1's selection of B2 or B3 in trial *n* is optimal, because the correct target has indeed switched. In this manner, the percentage of optimal choices is increased after M2 switch error (Supplementary Fig. 12). Note that owing to a failure of partner-action monitoring, M1 cannot distinguish between the causes of no-rewards in trial *n-1* (partner's choice error or switch error).
