# Supplementary material for: A causal role for frontal cortico-cortical coordination in social action monitoring
Source: Nat Commun. 2020 Oct 16;11:5233. doi: 10.1038/s41467-020-19026-y (PMC7568569; doi:10.1038/s41467-020-19026-y)
Supplement: Supplementary file 4 — Reporting Summary [file 41467_2020_19026_MOESM4_ESM.pdf]

nature research

Corresponding author(s): Masaki Isoda

Last updated by author(s): Sep 10, 2020

Reporting Summary

Nature Research wishes to improve the reproducibility of the work that we publish. This form provides structure for consistency and transparency in reporting. For further information on Nature Research policies, see Authors & Referees and the Editorial Policy Checklist.

Statistics

For all statistical analyses, confirm that the following items are present in the figure legend, table legend, main text, or Methods section.

n/a	Confirmed	
☐	☒	The exact sample size (*n*) for each experimental group/condition, given as a discrete number and unit of measurement
☐	☒	A statement on whether measurements were taken from distinct samples or whether the same sample was measured repeatedly
☐	☒	The statistical test(s) used AND whether they are one- or two-sided<br>*Only common tests should be described solely by name; describe more complex techniques in the Methods section.*
☐	☒	A description of all covariates tested
☐	☒	A description of any assumptions or corrections, such as tests of normality and adjustment for multiple comparisons
☐	☒	A full description of the statistical parameters including central tendency (e.g. means) or other basic estimates (e.g. regression coefficient) AND variation (e.g. standard deviation) or associated estimates of uncertainty (e.g. confidence intervals)
☐	☒	For null hypothesis testing, the test statistic (e.g. *F*, *t*, *r*) with confidence intervals, effect sizes, degrees of freedom and *P* value noted<br>*Give P values as exact values whenever suitable.*
☒	☐	For Bayesian analysis, information on the choice of priors and Markov chain Monte Carlo settings
☒	☐	For hierarchical and complex designs, identification of the appropriate level for tests and full reporting of outcomes
☐	☒	Estimates of effect sizes (e.g. Cohen's *d*, Pearson's *r*), indicating how they were calculated

Our web collection on statistics for biologists contains articles on many of the points above.

Software and code

Policy information about availability of computer code

Data collection	MATLAB 2016a (Mathworks Inc., Natick, MA, U.S.A.), MonkeyLogic MATLAB toolbox (Jan 5, 2017 build 47), Plexon OmniPlex data acquisition software (SortClient 1.15.1)
Data analysis	Statistics and machine learning toolbox (ver. 11.2), signal processing toolbox (ver. 7.5), parallel computing toolbox (ver. 6.11), control system toolbox (ver. 10.3), multivariate Granger causality toolbox (ver. 1.0) provided by MATLAB 2016a and 2017a (Mathworks Inc., Natick, MA, U.S.A.)

For manuscripts utilizing custom algorithms or software that are central to the research but not yet described in published literature, software must be made available to editors/reviewers. We strongly encourage code deposition in a community repository (e.g. GitHub). See the Nature Research guidelines for submitting code & software for further information.

Data

Policy information about availability of data

All manuscripts must include a data availability statement. This statement should provide the following information, where applicable:

- Accession codes, unique identifiers, or web links for publicly available datasets
- A list of figures that have associated raw data
- A description of any restrictions on data availability

The authors declare that the main data supporting the findings of this study are available within the paper and its supplementary information files. Extra data are available from the corresponding author upon reasonable request.

Field-specific reporting

Please select the one below that is the best fit for your research. If you are not sure, read the appropriate sections before making your selection.

☒ Life sciences ☐ Behavioural & social sciences ☐ Ecological, evolutionary & environmental sciences

For a reference copy of the document with all sections, see nature.com/documents/nr-reporting-summary-flat.pdf

Life sciences study design

All studies must disclose on these points even when the disclosure is negative.

Sample size	Behavioral and neural data were obtained from two monkeys (A and B). Two other monkeys (C and Q) were also used as nonrecorded participants. In total, activities of 565 single PMv neurons and 480 single MPFC neurons were recorded across 30 sessions. For pathway-selective intervention, we performed four courses of the Dox administration experiments in one monkey (B), during which behavioral data were collected across 66 (RA condition), 59 (FM condition), and 59 sessions (FO condition). Although no statistical methods were used to predetermine these sample sizes, they were similar to those reported in previous publications (Cf. Yoshida et al., Nature Neurosci 15: 1307-1312, 2012, ref #39; Waldert et al., J Neurosci 35: 8451-8461, 2015, ref #44; Kinoshita et al., Nature 487: 235-238, 2012, ref #37).
Data exclusions	For valid application of the Granger causality analysis, LFP time series data having problems of colinearity, nonstationarity, and/or heteroscedasticity were excluded in accordance with procedures established previously (Cohen. Analyzing neural time series data: theory and practice, 2014, ref #62; Barnett et al. J Neurosci Methods 223: 50-68, 2014, ref #63). The exclusion criteria were pre-established.
Replication	Two monkeys were used to verify the reproducibility. Reproducibility was confirmed.
Randomization	In the behavioral task, the correct target position was determined pseudorandomly. When testing the effect of the partner's biological nature, no experimental grouping was performed based on animals; both of the recorded monkeys were studied under all the partner conditions (i.e., RA, FM and FO conditions).
Blinding	Data collection and analysis were not performed blinded to the conditions of the experiments, because blinding was not possible due to requirements of experimenter controlled induction of protocols. However, we did not select the type of neurons during neural data acquisition; all well-isolated neurons were sampled. Moreover, the criteria used for data analyses were the same for different conditions and the analysis was performed automatically using Matlab scripts.

Reporting for specific materials, systems and methods

We require information from authors about some types of materials, experimental systems and methods used in many studies. Here, indicate whether each material, system or method listed is relevant to your study. If you are not sure if a list item applies to your research, read the appropriate section before selecting a response.

Materials & experimental systems

n/a	Involved in the study	
☐	☒	Antibodies
☒	☐	Eukaryotic cell lines
☒	☐	Palaeontology
☐	☒	Animals and other organisms
☒	☐	Human research participants
☒	☐	Clinical data

Methods

n/a	Involved in the study	
☒	☐	ChIP-seq
☒	☐	Flow cytometry
☒	☐	MRI-based neuroimaging

Antibodies

Antibodies used	GFP recombinant rabbit monoclonal antibody, Thermo Fisher Scientific, Catalog # G10362.<br>Biotinylated horse anti-rabbit IgG antibody, Vector Laboratories, Catalog # BA-1100.
Validation	For validation statements, relevant citations, and antibody profiles regarding the primary antibody, see: https://www.thermofisher.com/antibody/product/GFP-Antibody-Recombinant-Monoclonal/G10362.

Animals and other organisms

Policy information about studies involving animals; ARRIVE guidelines recommended for reporting animal research

Laboratory animals	Four male macaque monkeys [Macaca fuscata, designated as A (age 6), B (age 6), C (age 8) and Q (age 5)] were used as experimental subjects.

Wild animals	This study did not involve wild animals.
Field-collected samples	This study did not involve field-collected samples.
Ethics oversight	All animal care and experimentation protocols were approved by the Institutional Animal Care and Use Committee of National Institutes of Natural Sciences and were conducted in accordance with the guidelines described in the US National Institutes of Health Guide for the Care and Use of Laboratory Animals.

Note that full information on the approval of the study protocol must also be provided in the manuscript.
